# CXCL16/CXCR6/TGF‐β Feedback Loop Between M‐MDSCs and Treg Inhibits Anti‐Bacterial Immunity During Biofilm Infection

**DOI:** 10.1002/advs.202409537

**Published:** 2024-12-24

**Authors:** Xiaoyu Wu, Baiqi Pan, Chenghan Chu, Yangchun Zhang, Jinjin Ma, Yang Xing, Yuanchen Ma, Wengang Zhu, Huan Zhong, Aerman Alimu, Guanming Zhou, Shuying Liu, Weishen Chen, Xiang Li, Sheng Puyi

**Affiliations:** ^1^ Department of Joint Surgery The First Affiliated Hospital of Sun Yat‐sen University Guangzhou Guangdong 510080 China; ^2^ Guangdong Provincial Clinical Research Center for Orthopedic Diseases The First Affiliated Hospital of Sun Yat‐sen University Guangzhou Guangdong 510080 China; ^3^ Guangdong Provincial Key Laboratory of Orthopaedics and Traumatology Guangzhou Guangdong 510080 China; ^4^ Department of Orthopedics The People's Hospital of Baoan Shenzhen Shenzhen Guangdong 518101 China; ^5^ Department of Orthopedics The Second Affiliated Hospital of Shenzhen University Shenzhen Guangdong 518101 China; ^6^ Technology School of Medicine South China University of Technology Guangzhou Guangdong 510640 China; ^7^ Shien‐ming Wu School of Intelligent Engineering South China University of Technology Guangzhou Guangdong 510640 China; ^8^ Department of Orthopedics Guangdong Provincial People's Hospital (Guangdong Academy of Medical Sciences) Southern Medical University Guangzhou Guangdong 519041 China; ^9^ Department of Joint Orthopedics Yuebei People's Hospital Shaoguan Guangdong 512099 China; ^10^ Department of Joint Surgery Affiliated Hospital of Guangdong Medical University Zhanjiang Guangdong 524002 China; ^11^ Department of Orthopedics Foshan Hospital of Traditional Chinese Medicine Guangzhou Guangdong 528051 China; ^12^ Department of Histology and Embryology Zhongshan School of Medicine Sun Yat‐sen University Guangzhou Guangdong 510080 China; ^13^ Department of Spine Surgery The First Affiliated Hospital Sun Yat‐sen University Guangzhou Guangdong 510080 China

**Keywords:** Immunosuppression, M‐MDSCs, Periprosthetic joint infection, *Staphylococcus aureus*, Treg

## Abstract

*Staphylococcus aureus (S. aureus)* is a leading cause of Periprosthetic  joint  infection (PJI), a severe complication after joint arthroplasty. Immunosuppression is a major factor contributing to the infection chronicity of *S. aureus* PJI, posing significant treatment challenges. This study investigates the relationship between the immunosuppressive biofilm milieu and *S. aureus* PJI outcomes in both discovery and validation cohorts. This scRNA‐seq analysis of synovium from PJI patients reveals an expansion and heightened activity of monocyte‐related myeloid‐derived suppressor cells (M‐MDSCs) and regulatory T cells (Treg). Importantly, CXCL16 is significantly upregulated in M‐MDSCs, with its corresponding CXCR6 receptor also elevated on Treg. M‐MDSCs recruit Treg and enhance its activity via CXCL16‐CXCR6 interactions, while Treg secretes TGF‐β, inducing M‐MDSCs proliferation and immunosuppressive activity. Interfering with this cross‐talk in vivo using Treg‐specific CXCR6 knockout PJI mouse model reduces M‐MDSCs/Treg‐mediated immunosuppression and alleviates bacterial burden. Immunohistochemistry and recurrence analysis show that PJI patients with CXCR6^high^ synovium have poor prognosis. This findings highlight the critical role of CXCR6 in Treg in orchestrating an immunosuppressive microenvironment and biofilm persistence during PJI, offering potential targets for therapeutic intervention.

## Graphical abstract

In *S. aureus* biofilm‐associated PJI, M‐MDSCs recruit Treg via the CXCL16‐CXCR6 axis. Treg secrete TGF‐β, which promotes M‐MDSCs expansion and enhances their immunosuppressive function. This positive feedback loop intensifies immune suppression and contributes to refractory infections. CXCR6 has the potential to become a promising target for clinical treatment of PJI.

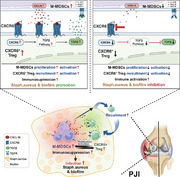

## 1 Introduction

Periprosthetic joint infection (PJI) remains a devastating complication after joint arthroplasty.^[^ ^1^, ^2^ ^]^ The incidence rate of PJI after total joint arthroplasty ranges from 1% to 2%.^[^ ^3^, ^4^ ^]^ With an increased number of patients requiring arthroplasties, higher incidence of PJI will occur in the future.^[^ ^5^, ^6^ ^]^ Debridement, antibiotics and implant retention, One‐stage and Two‐stage surgical revision strategies are the most common therapeutic options for PJI.^[^ ^7^, ^8^ ^]^ Despite undergoing above therapies, there are 15%–20% re‐infection rate in PJI patients.^[^ ^9^, ^10^ ^]^ Therefore, a novel therapeutic strategy of PJI is necessary to explore.

*Staphylococcus aureus (S. aureus)* is the primary pathogens of PJI,^[^ ^11^ ^]^ accounting for over 50% of cases,^[^ ^12^ ^]^ which is characterized by biofilm formation.^[^ ^13^, ^14^ ^]^ Biofilm, a kind of complicated microbial communities, tends to adhere to interfaces of prosthesis. It has reported that the formation of biofilm contributes to the development of PJI as a chronic and recurrent disease.^[^ ^15^ ^]^ Microorganisms typically comprise less than 10% of the dry mass in biofilms, whereas the matrix can constitute more than 90%. Within biofilms, microorganisms inhabit an environment formed by a self‐produced extracellular polymeric substances (EPS) matrix.^[^ ^16^ ^]^ EPS are primarily composed of polysaccharides, proteins, nucleic acids, and lipids. They contribute to the mechanical stability of biofilms, facilitate surface adhesion, and establish a cohesive, 3D polymer network that links and temporarily immobilizes bacteria in biofilms.^[^ ^17^ ^]^ The dense structure of biofilm on prostheses protects *S. aureus* from surrounding environmental stresses, impedes antibiotic penetration, and confers long‐term persistence capacity.^[^ ^18^, ^19^ ^]^ Moreover, the emergence of heterogeneous zones lacking nutrients and oxygen within biofilm causes bacteria to be in a dormant state, which render them resistant to antibiotics.^[^ ^20^, ^21^ ^]^ Collectively, the characteristics of biofilm decreased the effectiveness of antibiotic therapy, and then the bacterial infections will develop into chronic infections. Therefore, the innate and adaptive immune responses of host play an important role on inhibiting and eliminating the form of *S. aureus* biofilm. However, recent studies have found that *S. aureus* biofilm transfers host anti‐bacterial immune response into immunosuppressive phenotype that is easier for bacteria persistence.^[^ ^22^, ^23^, ^24^, ^25^, ^26^, ^27^ ^]^ Some studies have demonstrated that *S. aureus* biofilm infections correlate to the infiltration and function of Myeloid‐derived suppressor cells (MDSCs),^[^ ^24^, ^26^, ^28^, ^29^, ^30^, ^31^ ^]^ whereas the mechanisms are unclear.^[^ ^24^, ^30^, ^31^ ^]^

MDSCs are a subset of immature myeloid cells with vigorous immune‐suppressive activity involved in inhibition of anti‐bacterial response in many infections.^[^ ^32^, ^33^ ^]^ Various pathogens, particularly *S. aureus*, promote the expansion of MDSCs.^[^ ^34^ ^]^ The ability of MDSCs to dampen effector T‐cell responses contributes to their immunosuppressive nature, impacting the overall efficacy of the immune system.^[^ ^35^ ^]^ This, in turn, favors pathogen persistence and increases the risk of chronic infection. Furthermore, MDSCs facilitates the formation of biofilm by secreting interleukin‐10 (IL‐10), thereby inducing aggressive infections in PJI.^[^ ^26^ ^]^ Regulatory T cell (Treg) is another subset of immunosuppressive cells, playing an important role in maintaining immune system homeostasis and tolerance.^[^ ^36^, ^37^, ^38^ ^]^ The immunoregulatory actions of Treg have been proven to refer to *S. aureus* infection or sepsis.^[^ ^39^, ^40^, ^41^ ^]^ Meanwhile, recent works have indicated that Treg can regulate the differentiation of MDSCs and enhance their functions.^[^ ^39^, ^42^ ^]^ Although MDSCs and Treg are significant of *S. aureus* infection and biofilm formation, comparatively less is known about the overall microenvironment of PJI or how the cross‐talk between immune cells is affected by *S. aureus* biofilm in the context of PJI.

The CXCL16‐CXCR6 axis has been reported to be involved in regulation of immune response in many diseases,^[^ ^43^, ^44^, ^45^, ^46^ ^]^ because CXCL16 can induce the migration of CXCR6‐expressing leukocytes, which include CD8+ and CD4+ T cells, NK cells, invariant natural killer T cells, plasma cells, and monocytes. On the one hand, the CXCL16‐CXCR6 axis plays a pro‐inflammatory role in the pathogenic processes of various diseases, including lung infections, tumors, and autoimmune illnesses like experimental autoimmune encephalomyelitis (EAE), autoimmune hepatitis, rheumatoid arthritis (RA), and inflammatory bowel diseases (IBDs).^[^ ^47^ ^]^ In cases of CXCR6 deficiency, host control improved in pulmonary tuberculosis and influenza infection in the lung due to CXCR6's independence from T‐lymphocyte recruitment to the lungs.^[^ ^43^ ^]^ In EAE, the CXCL16‐CXCR6 axis recruits pathogenic CD4+ T cells to the central nervous system, exacerbating inflammation;^[^ ^48^ ^]^ in RA, the axis regulates T cell migration to the synovium and boosts interferon‐γ production;^[^ ^49^, ^50^ ^]^ while in IBDs, it promotes Th17 cell infiltration into the colonic mucosa, facilitating bacterial uptake by macrophages to enhance microbial inflammation, and can also activate pathways such as MAPK.^[^ ^51^, ^52^, ^53^ ^]^

On the other hand, it remains possible that the CXCL16‐CXCR6 axis also plays anti‐inflammatory roles in certain immune contexts.^[^ ^54^, ^55^ ^]^ The CXCL16‐CXCR6 axis is part of complex signaling loops that involve tumor cells, mesenchymal stem cells (MSCs), and immune cells. In one loop, CXCL16 secreted by breast cancer cells binds to CXCR6 on MSCs, driving MSCs recruitment. In turn, MSCs secrete chemokines that promote the recruitment of tumor‐associated macrophages and MDSCs, both of which are important in the immunosuppressive tumor microenvironment.^[^ ^54^ ^]^ Additionally, the axis plays a dual role in the tumor microenvironment. Initially, it facilitates T cell activation and promotes T cell migration to the tumor site, aiding the early immune response. However, once T cells infiltrate the tumor, this axis shifts toward immunosuppression, compromising T cell functionality and facilitating immune evasion within the tumor.^[^ ^55^ ^]^ Thus, CXCR6 has double‐edged effects on regulating immune response. Its function is different in different disease. As to *S. aureus*‐infected condition, previous studies have still not discussed its functions.

This study collected scRNA‐seq data from patients with *S. aureus* PJI, which systematically described an immunosuppressive profile of PJI microenvironment and indicated the cross‐talk effect between monocyte‐related myeloid‐derived suppressor cells (M‐MDSCs) and Treg. With *S. aureus* biofilm, M‐MDSCs secreted a large number of CXCL16 to recruit and strengthen Treg by recognizing CXCR6, while stimulated Treg also produced TGF‐β to enhance proliferation and immunosuppressive activity of M‐MDSCs. This was accomplished by engineering Treg‐specific CXCR6 knockout PJI mouse model. As expected, Treg infiltrating was significantly decreased, and the immunosuppressive activities of M‐MDSCs and Treg were impeded, contributing to immune‐mediated biofilm clearance and joint functional recovery. In summary, this study identified CXCR6 as a significant immune suppressor present on Treg, providing a potential target to reverse the immune suppression for PJI immunotherapy, thus enhancing effect of revision surgery and antibiotic.

## 2 Results

### 2.1 A Large‐Scale Single‐Cell Atlas of Patients with PJI

**Figure 1:**
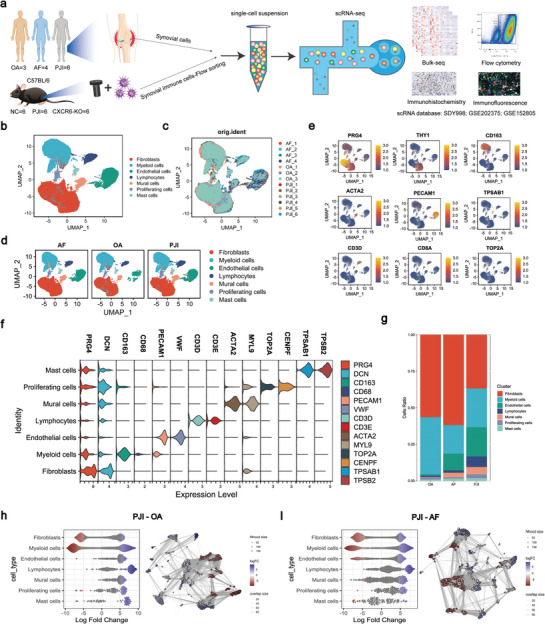
The single‐cell landscape of PJI, AF and OA knee synovial tissues. a) Schematic illustration of the experimental strategy. The human knee joint synovium scRNA‐seq data was utilized as a discovery cohort. b) Atlas of 151185 single cells collected from 6 PJI, 4 AF and 3 OA knee synovial tissues, displaying seven major cell types by Uniform Manifold Approximation and Projection (UMAP) plot. c,d) The UMAP plot showed commonalities of each sample and different groups after correcting for batch effects by Harmony. e,f) The violin plot and UMAP plot showed the expression level of biomarkers for major cell types. g, The bar chart showed the proportion of each cell type in PJI, AF and OA. The color panel indicated different cell types in the scRNA‐seq data. h,i) Milo analysis revealed differential abundance of cell neighborhoods in PJI versus AF and PJI versus OA in synovial cell subpopulations.

To elucidate the cellular landscape of knee *S. aureus* PJI patients’ synovial tissues, we performed scRNA‐seq analysis on synovial tissues obtained from 6 patients with PJI, while 4 aseptic failure (AF) and 3 osteoarthritis (OA) patients’ synovial tissues were included as controls. The demographics of the 13 subjects included in this study were presented in **Table** **1**. All the PJI patients were infected with *S. aureus* as the pathogenic bacteria. OA patients were incorporated as controls to facilitate a comparative analysis of the distinctions between non‐infected synovitis and *S. aureus*‐infected synovitis. We also selected AF patients undergoing aseptic revision as controls to exclude interference from the prosthesis. In order to corroborate our findings from the scRNA data, we employed immunohistochemistry (IHC) (PJI:AF:OA = 80:25:25) and bulk RNA‐sequencing techniques (PJI:AF = 18:18) as the two validation cohorts. Additionally, we also utilized publicly available sequencing data of knee joint synovial scRNA‐seq data in ImmPort and Gene Expression Omnibus (GEO) database to establish the public validation cohorts (**Figure** **1**a). The analysis of scRNA results identified a total of 134756 cells resulting in seven clusters from the 13 patients, with PJI, AF, and OA, accounting for 53765, 47659 and 33332 cells respectively. The clusters were identified as specific cell subpopulations based on the expression of known cell type‐specific markers and cluster‐specific genes, including fibroblasts (THY1, PRG4 and COL1A1), mural cells (ACTA2 and CD248), lymphocytes (CD3D, CD3E and CD4), myeloid cells (CD163, CD68, CD14), endothelial cells (PECAM1, VWF and CD34), mast cells (TPSAB1 and TPSB2), and proliferating cells (TOP2A, MKI67 and ZNF367) (Figure 1b,e,f; Figure S1e,f, Supporting Information). As human neutrophils are extremely delicate and vulnerable in vitro,^[^ ^56^ ^]^ they have been hardly detected in most of previous single‐cell studies of human synovial tissue of knee joint using 10x Genomics Chromium platform.^[^ ^57^, ^58^, ^59^ ^]^ In our study, a few neutrophils were captured in only two PJI samples, and these results did not display. After the removal of batch effects, all samples were integrated and exhibited identical cell type compositions, indicating a low degree of individual heterogeneity among the samples (Figure 1c,d; Figure S1a, Supporting Information). All subpopulations were shared among PJI samples in similar proportions and numbers (Figure 1g; Figure S1b–d, Supporting Information). Except for fibroblasts, myeloid cells accounted for the largest proportion in all samples (Figure 1g). Furthermore, the PJI patients exhibited a significantly differential abundance of myeloid cells and lymphocytes in the knee joint synovium (Figure 1h,i). Therefore, we conducted further analysis on these two cell subtypes to delineate the characteristic immune profiles of *S. aureus* PJI.

**Table 1:** Characteristics of the PJI, AF, and OA.

	PJI_1	PJI_2	PJI_3	PJI_4	PJI_5	PJI_6	AF_1	AF_2	AF_3	AF_4	OA_1	OA_2	OA_3
Sex(F/M)	F	M	F	M	M	F	F	M	F	F	F	M	F
Age	72	77	88	70	72	62	69	67	74	43	51	52	69
Time of implantation(months)	4	3	48	6	12	9	168	60	96	60	Not Applicable	Not Applicable	Not Applicable
Microorganisms	S.aureus	S.aureus	S.aureus	S.aureus	S.aureus	S.aureus	/	/	/	/	/	/	/
Indication (primary arthroplasty)	Osteoarthritis	Osteoarthritis	Osteoarthritis	Osteoarthritis	Osteoarthritis	Osteoarthritis	Osteoarthritis	Osteoarthritis	Osteoarthritis	Osteoarthritis	Osteoarthritis	Osteoarthritis	Osteoarthritis
Manufacturer (primary arthroplasty)	Smith&Nephew	Smith&Nephew	LINK	Smith&Nephew	ZIMMER	LINK	ZIMMER	Depuy	Smith&Nephew	Smith&Nephew	Smith&Nephew	ZIMMER	LINK
Material type (primary arthroplasty)	Titanium alloy	Titanium alloy	Titanium alloy	Titanium alloy	Titanium alloy	Titanium alloy	Titanium alloy	Titanium alloy	Titanium alloy	Titanium alloy	Titanium alloy	Titanium alloy	Titanium alloy
Coating (primary arthroplasty)	Hydroxyapatite	Hydroxyapatite	Hydroxyapatite	Hydroxyapatite	Hydroxyapatite	Hydroxyapatite	Hydroxyapatite	Hydroxyapatite	Hydroxyapatite	Hydroxyapatite	Hydroxyapatite	Hydroxyapatite	Hydroxyapatite
CRP (mg/L)	35.53	28.73	114.47	74.94	55.65	61.84	2.65	1.02	4.25	4	3.6	2.58	3.57
ESR (mm/h)	89	76	110	108	93	111	40	26	27	13	8	5	10
Kellgren‐Lawrence grade	Not Applicable	Not Applicable	Not Applicable	Not Applicable	Not Applicable	Not Applicable	Not Applicable	Not Applicable	Not Applicable	Not Applicable	IV	IV	IV
Krenn score	Not Applicable	Not Applicable	Not Applicable	Not Applicable	Not Applicable	Not Applicable	Not Applicable	Not Applicable	Not Applicable	Not Applicable	2	1	2

### 2.2 The Expanded and Activated M‐MDSCs in the Immunosuppressive Environment of PJI

**Figure 2:**
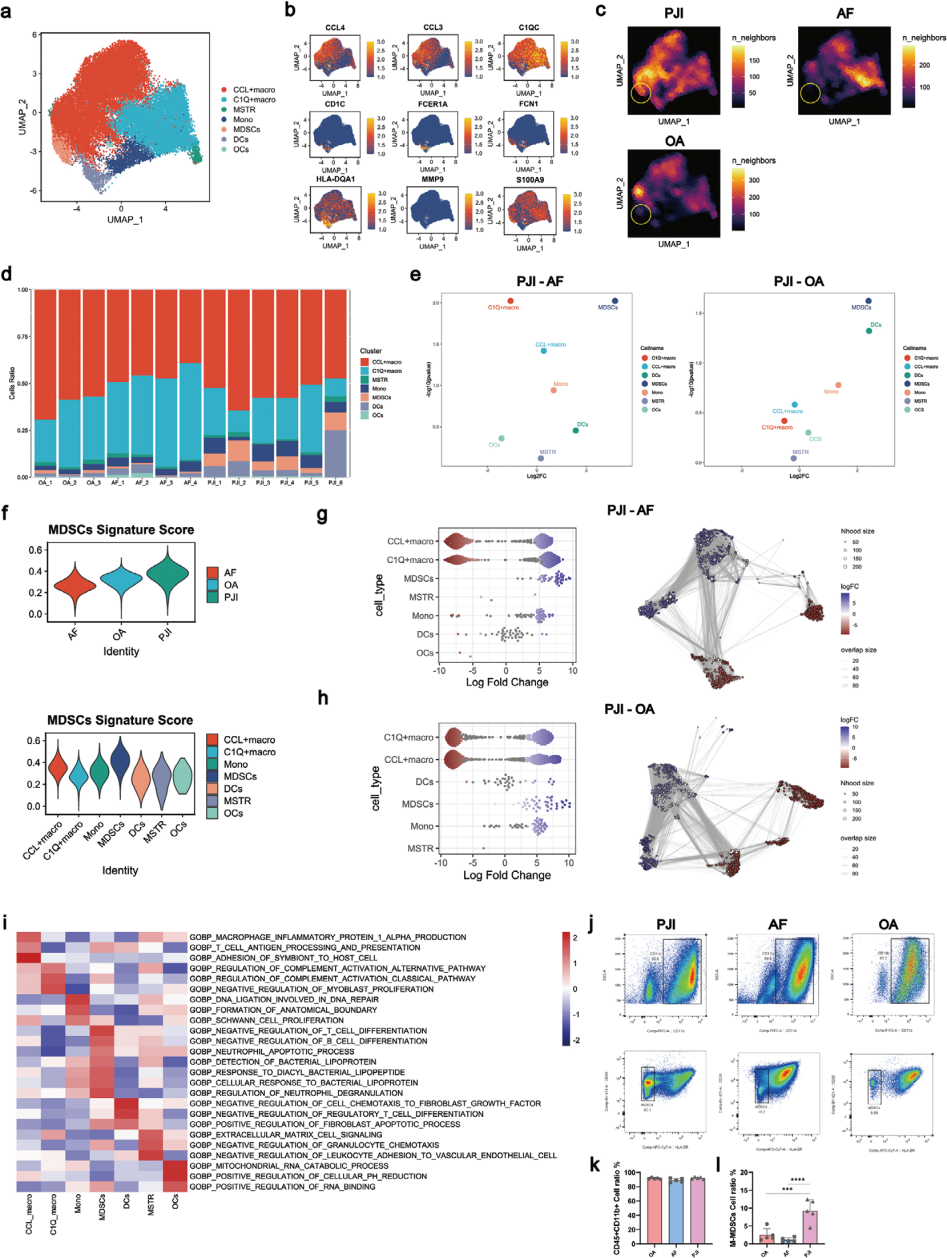
PJI samples exhibited increased infiltration of M‐MDSCs accompanied by enhanced immunosuppressive activity. a) The UMAP plot showed detailed annotation of the myeloid subpopulations after re‐clustering. Seven myeloid clusters were visualized. Macro: Macrophage, Mono: Monocyte, MSTR: Monocyte‐stress response, DCs: Dendritic cells, OCs: Osteoclasts. b) The UMAP plot showed the expression level of biomarkers for myeloid cell subpopulations. c) Changes in the composition of the myeloid compartment among PJI, AF and OA were visualized as cell density. d) The bar chart showed the proportion of each myeloid subpopulation in different samples (PJI, n = 6; AF, n = 4; OA, n = 3). e) The scatter plot compared the relative abundance of myeloid cell subpopulations in PJI versus AF and OA. The x and y axes represented the log2 fold change and p‐value, respectively, based on the Mann‐Whitney U test. Each dot represented a specific cell type. f) Violin plots showed the MDSCs scores in seven myeloid subpopulations, as well as in PJI, AF and OA. g,h) Milo analysis revealed differential abundance of cell neighborhoods in PJI versus AF and PJI versus OA in the myeloid subpopulations. i) Gene set variation analysis (GSVA) showed different enriched pathways of the seven myeloid subpopulations. j) The flow cytometry plots depicted the gating strategy employed to identify M‐MDSCs derived from PJI samples (left), AF samples (middle), and OA samples (right). Percentages were calculated over target populations. k,l) Quantitative analysis of M‐MDSCs and myeloid cells in PJI, AF, and OA (PJI, n = 5; AF, n = 5; OA, n = 5). Unpaired t test; ***p < 0.001, ****p < 0.0001.

The myeloid cells, acting as the primary defense against bacterial infection in the synovium, are believed to play a crucial role in the development of PJI.^[^ ^60^ ^]^ After re‐clustering, our results revealed the presence of seven distinct subpopulations in myeloid cells, including C1Q+myelo (C1QA, C1QB and C1QC), CCL+myelo (CCL3, CCL4 and TNF), MSTR (HSPS1, HSPD1 and HSPA1A), Mono (TMP1 and HCST), MDSCs (S100A8, S100A9 and FCN1), DCs (FCER1A, CD1C, HLA‐DQA1), OCs (MMP9, ACP5 and NFATC1) (**Figure** **2**a,b; Figure S2b, Supporting Information). The proportion of MDSCs significantly increased in PJI group compared to AF group and OA group (Figure 2c–e; Figure S2a, Supporting Information). The transcriptomic analysis revealed robust immunomodulatory effects of MDSCs, as evidenced by the upregulation of calprotectin (S100A8 and S100A9) and interleukins (IL1B and IL10) (Figure S2c, Supporting Information).^[^ ^61^ ^]^ We subsequently employed the MDSCs gene signature^[^ ^62^ ^]^ to evaluate the gene scoring of all myeloid cell subpopulations, confirming that MDSCs cluster we identified exhibited the highest score than others (Figure 2f). To further investigate the function of M‐MDSCs, we employed GSVA analysis on seven distinct myeloid subpopulations. Our findings revealed that MDSCs were significantly enriched in gene sets associated with immunosuppression, such as “Negative regulation of T cell differentiation”, “Negative regulation of B cell differentiation”, “Neutrophil apoptotic process”, and “Detection of bacterial lipoprotein” (Figure 2i). Above results confirmed that MDSCs cluster identified in our study exhibited gene signature and biological functions were consistent with the characteristics of MDSCs reported in other studies, thus validating the accuracy of our classification of the MDSCs cluster.^[^ ^63^, ^64^ ^]^ As we know, depending on the origin, MDSCs have been classified into two subsets: Polymorphonuclear myeloid‐derived suppressor cells (PMN‐MDSCs) and M‐MDSCs. PMN‐MDSCs mainly suppressed non‐specific immune responses^[^ ^65^ ^]^ and M‐MDSCs suppressed both antigen‐specific and non‐specific immune responses. Therefore M‐MDSCs had stronger immunosuppressive activity than PMN‐MDSCs.^[^ ^66^, ^67^ ^]^ In order to confirm the identified subtype of MDSCs in our study, we initially conducted an analysis of characteristic markers associated with MDSCs. The results indicated that MDSCs in our study were highly expressed CD11b, CD33, and CD14, and seldom expressed HLA‐DR, which were typical features of monocytes‐derived cells (Figure S2f, Supporting Information). Second, the neutrophils of two patients were incorporated with myeloid subpopulations to perform re‐clustering analysis, which showed a distance between neutrophils subpopulation and MDSCs subpopulation in the UMAP plot, and MDSCs subpopulation did not expressed neutrophil‐specific markers, such as FCGR3B, CSF3R (Figure S2d,e, Supporting Information). Therefore, we determined that the MDSCs in our study were M‐MDSCs originating from monocytes. Importantly, compared to the myeloid cell subpopulations in AF group and OA group, the differential abundance of genes in M‐MDSCs were significantly increased in PJI group, suggesting that M‐MDSCs were more momentous subpopulations in PJI(Figure 2g,h). In addition, M‐MDSCs expressed a significantly higher number of immunosuppressive genes and were enriched in more immunosuppressive pathways in PJI, when compared to the AF and OA groups (Figure S2f,g, Supporting Information). To validate the augmented levels of M‐MDSCs in PJI by scRNA analysis, we performed flow cytometry on knee synovial cells from PJI, OA, and AF patients. The results revealed the ratio of myeloid cells were similar among the three groups, however, an increasing number of M‐MDSCs were observed in the PJI group (*p* < 0.01) (Figure 2j–l). The collective evidence suggested that in the synovium of PJI, the quantity and immunosuppressive activity of M‐MDSCs were dramatically increased, accounting for immunosuppressive environment in PJI.

### 2.3 The Increased Treg in PJI Suppressed Lymphocytes Activity

**Figure 3:**
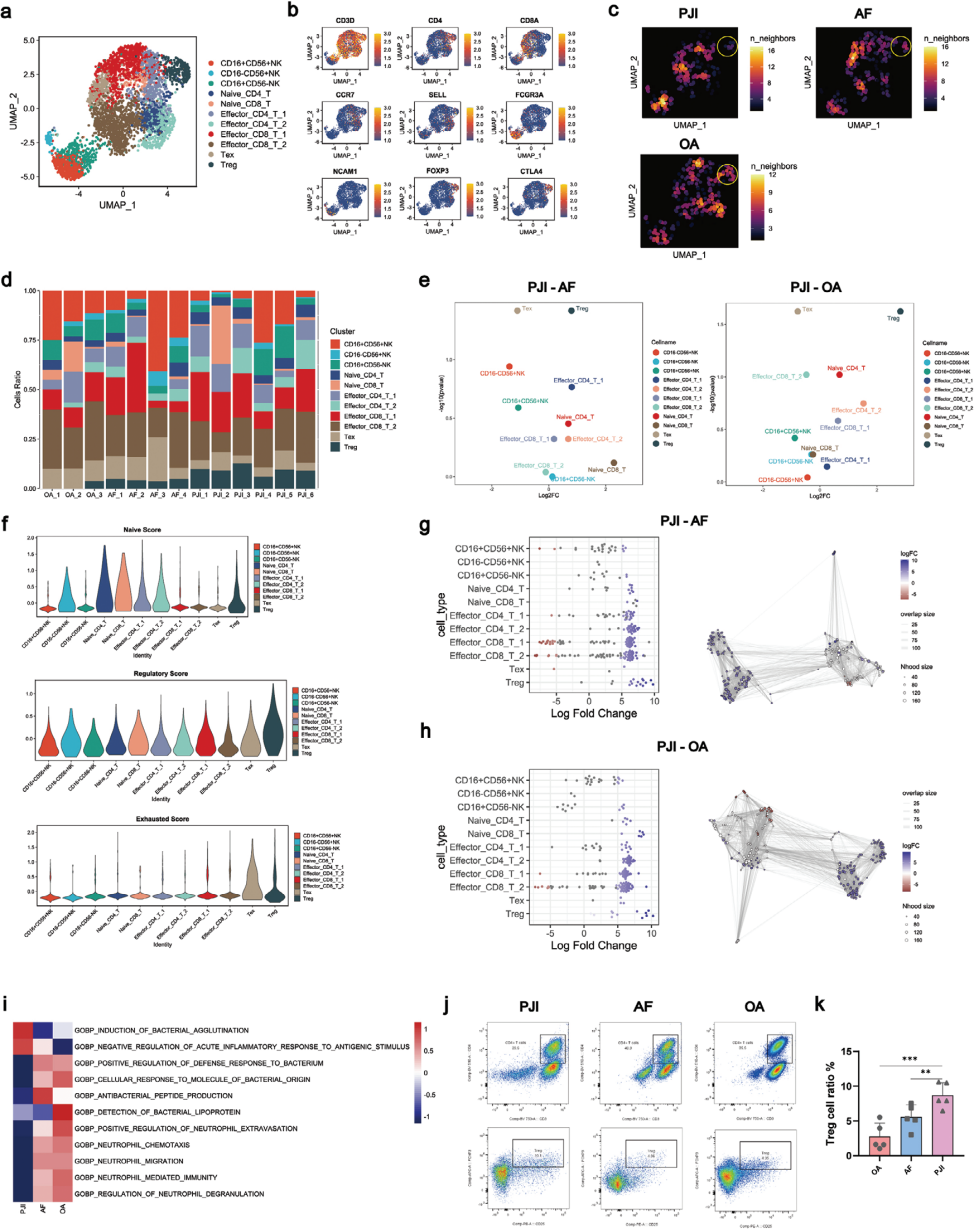
PJI samples exhibited increased infiltration of Treg and low‐activated phenotype of other lymphocytes. a) The UMAP plot showed detailed annotation of the lymphocytes subpopulations after re‐clustering. Eleven lymphocytes clusters were visualized. Tex: Exhaust T cell, Treg: Regulatory T cell. b) The UMAP plot showed the expression level of biomarkers for lymphocytes subpopulations. c) Changes in the composition of the lymphocytes compartment among PJI, AF and OA were visualized as cell density. d) The bar chart showed the proportion of each lymphocyte subpopulation in different samples (PJI, n = 6; AF, n = 4; OA, n = 3). e) The scatter plot compared the relative abundance of lymphocytes subpopulations in PJI versus AF and OA. The x and y axes represented the log2 fold change and p‐value, respectively, based on the Mann‐Whitney U test. Each dot represented a specific cell type. f) Violin plots showed the naive, exhausted and regulatory scores of eleven lymphocytes subpopulations. g,h) Milo analysis revealed differential abundance of cell neighborhoods in PJI versus AF and PJI versus OA in the lymphocytes subpopulations. i) GSVA showed immune‐related signaling pathways of the lymphocytes subpopulations PJI versus AF, and PJI versus OA. j) The flow cytometry plots depicted the gating strategy employed to identify Treg derived from paired PJI samples (left), AF samples (middle), and OA samples (right). Percentages were calculated over target populations. k) Quantitative analysis of Treg in PJI, AF, and OA (PJI, n = 5; AF, n = 5; OA, n = 5). Unpaired t test; **p < 0.01, ***p < 0.001.

Lymphocytes were subjected to eleven subpopulations: naive CD8 T cells (Naive_CD8_T), naive CD4 T cells (Naive_CD4_T), exhausted T cells (Tex), regulatory T cells (Treg), three subpopulations of natural killer cells (CD16+CD56+NK, CD16‐CD56+NK, CD16+CD56‐NK), two subpopulations of effector CD4 T cells (Effector_CD4_T_1, Effector_CD4_T_2), and two subpopulations of effector CD8 T cells (Effector_CD8_T_1, Effector_CD4_T_2) (**Figure** **3**a,b). The results demonstrated a significant increase of Treg in the PJI group compared to the AF and OA groups (Figure 3c–e; Figure S3a, Supporting Information). Treg was characterized by high expression of multiple immunosuppressive genes (FOXP3, CTLA4, IL2RA), significantly elevated regulatory score and immunosuppressive function (Figure 3b,f; Figure S3b, Supporting Information). Trajectory analysis revealed that Treg was positioned at the terminal stage of the differentiation trajectory (Figure S3c,d, Supporting Information). Next, we conducted Milo analysis to examine lymphocyte subpopulations and found that Treg exhibited a significantly higher differential abundance of genes compared with other lymphocyte subpopulations, suggesting that it was the most prominently affected cell subpopulation by *S. aureus* biofilm in PJI environment. (Figure 3g,h). It has been reported that Treg is essential for suppressing sterilizing immunity. Treg can quickly expand and persist during chronic infection, thereby impeding the onset of cellular immunity.^[^ ^68^ ^]^ GSVA analysis of lymphocytes among PJI, AF and OA group showed significant downregulation of immune activation‐related pathways in the PJI group, including “Positive regulation of neutrophil extravasation,” “Production of antibacterial peptides,” and “Cellular response to molecules of bacterial origin” (Figure 3i). It suggested the overall suppression of lymphocyte functions in PJI was possibly associated with the increased Treg. To further confirm the increased quantity of Treg as indicated by scRNA‐seq analysis, flow cytometry assays were subsequently performed on knee synovial cells (Figure 3j). As expected, the number of Treg was found to be significantly higher in PJI compared to AF and OA patients (Figure 3k). The similar results were validated in the public database. The SDY998 dataset (OA, n = 4; RA, n = 20) (Figure S4f–n, Supporting Information) was obtained from the ImmPort database. The GSE202375 dataset (RA, flow sorting) (Figure S4a–e, Supporting Information) and the GSE152805 dataset (OA, n = 3) (Figure S4o–r, Supporting Information) were obtained from the GEO database. The proportion of Treg in PJI was also increased compared to OA and RA across multiple databases (Figure S4s, Supporting Information). Collectively, these findings implied that under the biofilm milieu of *S. aureus* PJI patients, the infiltration of Treg were prominently increased, which resulted in a limitation of sterilizing immunity.

### 2.4 The Expanded THY1+Fibro and Tip Cell in PJI

We also strictly re‐clustered non‐immune cells, including fibroblasts and endothelial cells. Fibroblasts were classified into four distinct subpopulations: PRG4+Fibroblasts (PRG4+Fibro), THY1+Fibroblasts (THY1+Fibro), CXCL12+Fibroblasts (CXCL12+Fibro), and POSTN+Fibroblasts (POSTN+Fibro) (Figure S5a,b, Supporting Information). The synovium of the knee joint was a double‐layered structure. PRG4 was predominantly expressed in the lining layer (LL), while THY1 was primarily expressed in the sublining layer, which is consistent with previous findings^[^ ^69^, ^70^, ^71^ ^]^ (Figure S5d–g, Supporting Information). PJI was characterized by the increase of THY1+Fibro, which was enriched in pathways of promoting vascular proliferation and fibroblast migration (Figure S5c,h, Supporting Information). The endothelial cells were classified into six subpopulations, including Vein (Vein_1, Vein_2 and Vein_3), Artery, Lymphatic, and Tip cell (Figure S6a,b, Supporting Information). The presence of Tip cell in PJI showed a significant increase (Figure S6c, Supporting Information). The Tip cell, characterized by the expression of PGF and PXDN, played a crucial role in promoting angiogenesis^[^ ^72^, ^73^ ^]^ (Figure S6f,g, Supporting Information). The trajectory analysis revealed that Tip cell had the capacity to differentiate into venous endothelial cell (Figure S6d–f, Supporting Information). The GSVA enrichment analysis suggested a potential association between Tip cell and the proliferation of mast cell and vascular endothelial cell (Figure S6h, Supporting Information). The above results indicated that PJI was also characterized by the increase of THY1+Fibro and Tip cell.

### 2.5 CellChat Reveal Cell‐Cell Interaction and the Mechanism of Treg Recruitment

**Figure 4:**
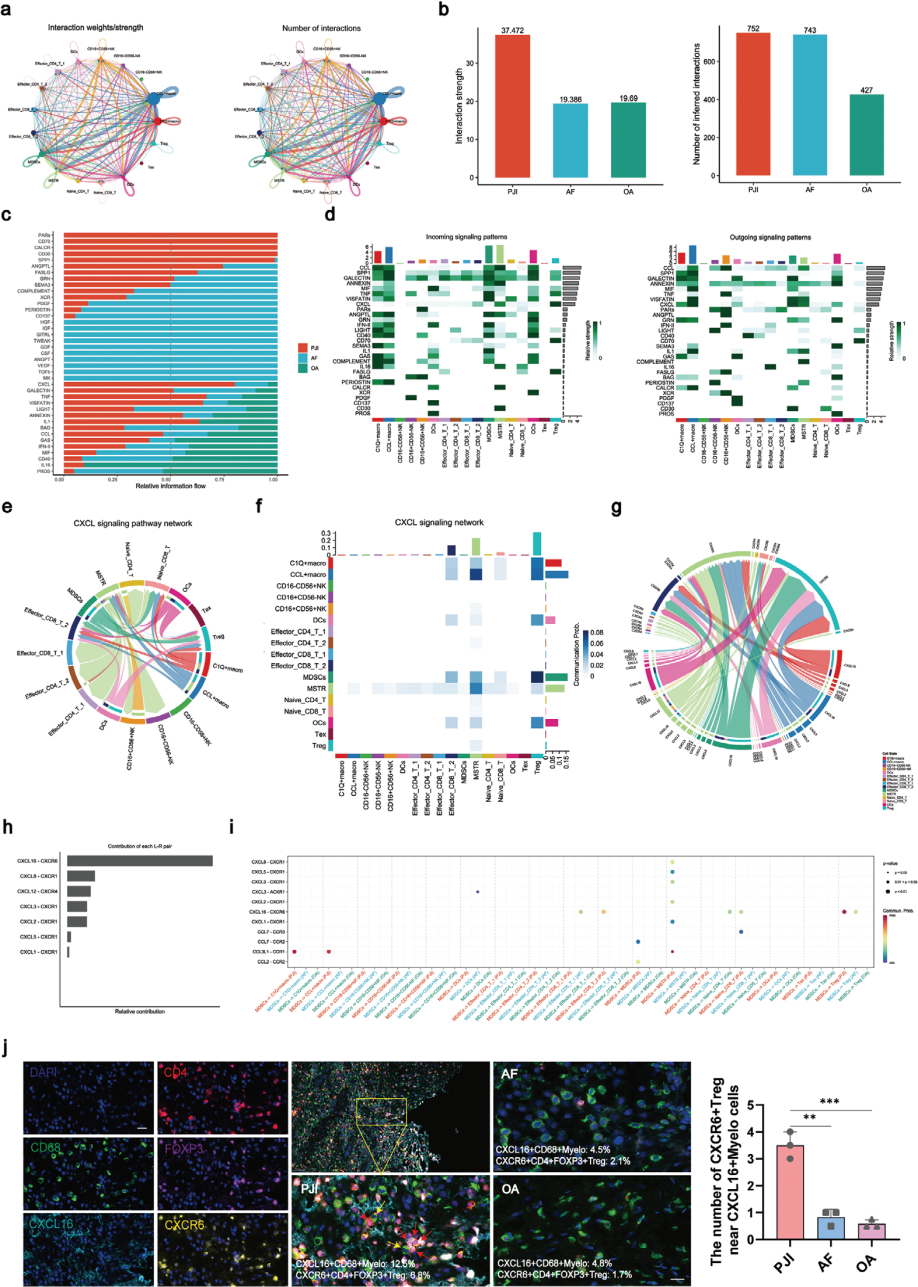
The interaction of MDSCs and Treg by CXCL16‐CXCR6 signaling was obvious in PJI. a) The interaction plot showed the cell communications between myeloid cells and lymphocytes by ligand‐receptor pair analysis. The thicker the line represented, the stronger the interaction weights/strength, and the more the number of interactions among the cell types. b) The bar chart showed the interaction strength and the number of interactions among PJI, AF and OA. c) The bar chart showed the strength comparison of the interaction pathways among PJI, AF and OA. d) The heatmap showed relative strength of all enriched signals (outgoing and incoming) across myeloid cells and lymphocytes. e) The circle plot showed the inferred intercellular communication network for CXCL signaling pathway. f) The heatmap showed the CXCL signaling pathway among myeloid cells and lymphocytes. g) The bar chart showed the ligand‐receptor pair of CXCL signaling pathway among myeloid cells and lymphocytes. h) The bar chart showed the relative contribution of ligand‐receptor pair to the overall communication network of CXCL signaling pathway. i) The dot plot showed the incoming communication patterns of CXCL signaling pathway from M‐MDSCs among PJI, AF and OA. j) Multiplex immunofluorescence image showed the co‐location of CXCR6+ Treg and CD68+ myeloid cells with excretive CXCL16. Yellow arrow highlighted the CD68+ myeloid cells expressing CXCL16, and red arrow highlighted the CD4+FOXP3+ Treg expressing CXCR6. Scale bar, 20 mm (PJI, n = 3; AF, n = 3; OA, n = 3). Unpaired t test; **p < 0.01, ***p < 0.001.

The above results showed that the immunosuppression related cells, including M‐MDSCs and Treg were significantly increased in *S. aureus* PJI. To understand the mechanism of immunosuppressive microenvironment remodeling, we established a comprehensive cell‐cell communication network among all immune cell subpopulations. Circle plots were used to visualize the number of interactions and the interaction strength based on ligand‐receptor pairs integrated from PJI, AF, and OA groups (**Figure** **4**a). The number of interactions and the interaction strengths in each group were summarized, indicating that there was a higher abundance of interaction within the PJI group (Figure 4b). By comparing the strength of different pathways among the three groups, the results found that PARs, CD70, CALCR, CD30, SPP1, ANGPTL, FASLG, CXCL, TNF, VISFATIN and IL1 pathways were upregulated in PJI group (Figure 4c). A total of 28 pathways associated with lymphocytes and myeloid cell interactions were detected in PJI group. Among them, the CXCL signaling pathway exhibited the highest relative strength index in incoming signaling patterns in Treg and outgoing signaling patterns in M‐MDSCs (Figure 4d). Network centrality analysis and heatmap visualization of inferred CXCL signaling network revealed a strong inter‐association between M‐MDSCs and Treg (Figure 4e,f). Ligand‐receptor analysis demonstrated that the CXCL16‐CXCR6 pairs made significant relative contributions to these interactions (Figure 4g–i; Figure S7a–g, Supporting Information). These results indicated that M‐MDSCs recruit Treg by CXCL16‐CXCR6 pairs in PJI. Subsequently, we investigated the expression of CXCL16 and CXCR6 in the myeloid cell and lymphocyte subpopulations among PJI, AF and OA group. The expression of CXCL16 was most significantly upregulated in M‐MDSCs of PJI group, while it also increased in other myeloid subpopulations of PJI group. On the other hand, the expression of CXCR6 was specifically upregulated in Treg of PJI group, while it seldom expressed in other lymphocyte subpopulations of PJI, AF and OA group (Figure S7h,I, Supporting Information).

To further verify the recruitment effect of CXCL16/CXCR6 axis on Treg, we conducted multiplex immunofluorescence staining to indicate the co‐location of CXCL16‐expressing myeloid cells (CD68+) and CXCR6‐expressing Treg (CD4+FOXP3+) in PJI group, not in AF and OA groups (Figure 4j). Importantly, the ELISA result showed that CXCL16 was predominantly expressed in M‐MDSCs, rather than PMN‐MDSCs (Figure S7j, Supporting Information). After co‐culturing the M‐MDSCs with the *S. aureus* biofilm, M‐MDSCs produced increased number of CXCL16 (Figure S7k, Supporting Information). In summary, under the *S. aureus* biofilm milieu, M‐MDSCs were predicted to recruit Treg through CXCL16‐CXCR6, leading to an immunosuppressive microenvironment.

### 2.6 PJI Patients with High CXCR6 Expression have a High Recurrence Rate

**Figure 5:**
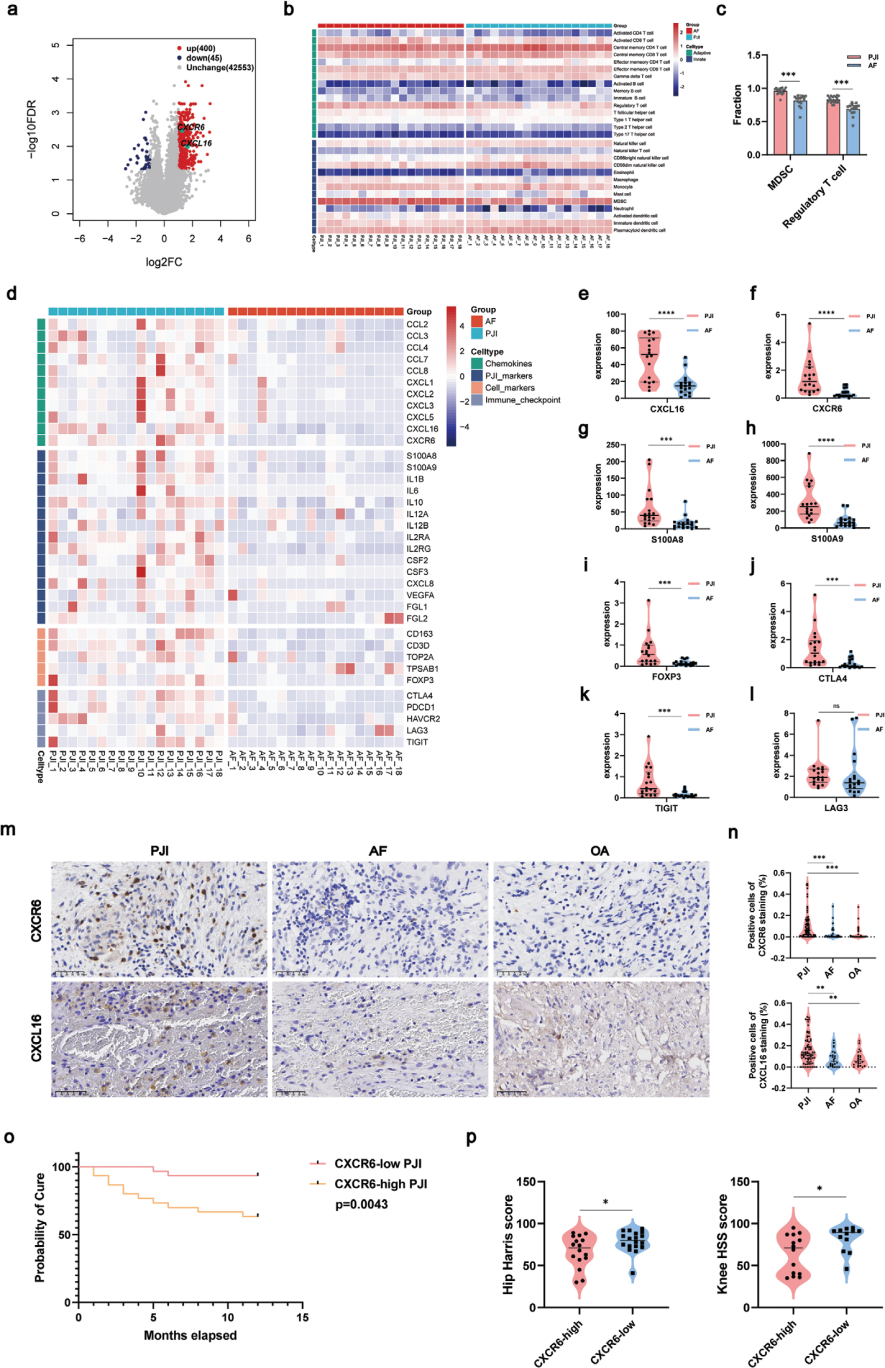
PJI patients with high CXCR6 showed a higher recurrence rate. a) The Volcano plot showed differentially expressed genes in PJI versus AF patients. The x and y axes represented the log2 fold change and p‐value, respectively, based on the Mann‐Whitney U test. Red dots: upregulated genes, blue dots: downregulated genes. b) The heatmap showed the distribution of 28 types of immune cells in PJI and AF. c) The bar chart showed the difference in proportion of M‐MDSCs and Treg between PJI and AF (PJI, n = 18; AF, n = 18). Unpaired t test; ***p < 0.001. d) The heatmap showed the characteristic genes of chemokines, PJI markers, cell markers and immune checkpoint between PJI and AF. e–l) The violin plots showed part of the differential genes (CXCL16, CXCR6, S100A8, S100A9, FOXP3, CTLA4, TIGIT and LAG3) between PJI and AF as shown in (d). Unpaired t test; ^ns^ *p* > 0.05, ***p < 0.001, ****p < 0.0001. m,n) Quantitative analysis of immunohistochemical staining for CXCL16 and CXCR6 (PJI, n = 80; AF, n = 25; OA = 25). 40x magnification. Scale bar, 20 mm. Unpaired t test; **p < 0.01, ***p < 0.001. o) The Kaplan‐Meier curve showed the recurrence curves of PJI patients characterized by either low or high expression of CXCR6 (CXCR6‐high PJI, n = 30; CXCR6‐low PJI, n = 30). Significance was calculated using the log‐rank test. p,q) Quantitative analysis of Harris Hip score and HSS knee score in CXCR6‐high PJI and CXCR6‐low PJI group (CXCR6‐high PJI, n = 30; CXCR6‐low PJI, n = 30). Unpaired t test; *p < 0.05.

The bulk‐seq analysis was performed on 36 knee synovium tissues of PJI and AF patients as the validation cohort. In comparison to AF patients, 400 up‐regulated and 45 down‐regulated differentially expressed genes (DEGs) were identified in the PJI patients. Among the upregulated genes, we highlighted CXCL16‐CXCR6 (**Figure** **5**a), which aligns with the significant conclusions drawn from single‐cell sequencing using CellChat. Additionally, the genetic expressions of chemokines, immune checkpoints, and infection‐related genes were increased in the PJI group (Figure 5d). The expression levels of CXCL16, CXCR6, S100A8/A9, FOXP3, CTLA4 and TIGIT were significantly upregulated in PJI samples of this validation cohort (Figure 5e–l). All of them are consistent with our scRNA‐seq conclusion (Figure 5a). To further investigate the differences in infiltrated immune cells between the PJI and AF samples, their relationship was assessed by the ssGSEA algorithm. The heatmap visualization of 28 immune cells indicated a significantly higher infiltration of Treg and MDSCs in PJI samples, which was consistent with our previous findings (Figure 5b,c). These findings supported our previous results, indicating that PJI patients exhibit increased expression of the CXCL16/CXCR6 axis and infiltration of Treg and M‐MDSCs.

Regarding the result of IHC staining of 130 knee synovial tissues, it was confirmed that CXCR6 and CXCL16 expression was significantly higher in the PJI group compared to both the AF group (CXCR6, p = 0.0007; CXCL16, p = 0.0049) and OA group (CXCR6, p = 0.0002; CXCL16, p = 0.0030) (Figure 5m,n). The PJI patients were subsequently classified into CXCR6‐high subgroup and CXCR6‐low subgroup based on the expression level of CXCR6 identified by IHC. There were no statistically significant differences in the clinical characteristics of these patients (**Table** **2**). The cure rate in the CXCR6_high PJI group was significantly lower compared to the CXCR6_low PJI group (p = 0.0043) (Figure 5o). The diagnostic criteria reference for recurrence of PJI referred to 2018 PJI Philadelphia International Consensus diagnostic criteria (**Table** **3**).^[^ ^74^ ^]^ In addition, the knee joint KSS score and hip joint Harris score also indicated a functional loss for CXCR6_high PJI patients (Figure 5p). In summary, we proved the increase of M‐MDSCs and Treg with the activation of CXCL16 and CXCR6 in two validation cohorts by bulk‐seq analysis and the IHC staining. Furthermore, our findings indicated a positive correlation between high levels of CXCR6 expression and poor prognosis of PJI.

**Table 2:** Baseline demographic of the CXCR6‐high PJI and CXCR6‐low PJI group.

	CXCR6‐high PJI	CXCR6‐low PJI	Statistical	*P* value
Age in years	77.0±11.9	75.2±12.2	*t* = 0.589	0.558^a)^
BMI in kg/m^2^	27.4 (22.6,29.3)	27.8(23.2,29.7)	*Z* = −0.458	0.647^b)^
Sex (male/female)	16/14	13/17	*χ^2^* = 0.601	0.438^c)^
Hypertension, %(n)	26.7% (8)	20.0% (6)	*χ^2^* = 0.373	0.542^c)^
coronary heart disease, %(n)	16.7% (5)	13.3% (4)	*χ^2^ =* 0	1.000^c)^
Diabetes, %(n)	30.0% (9)	16.7% (5)	*χ^2^ =* 1.491	0.222^c)^

^a)^ *P* value calculated using the student's t test;

^b)^ *P* value calculated using the Mann‐whitney U test;

^c)^ *P* value calculated using the chi‐square test BMI, body mass index

**Table 3:** Diagnostic criteria of periprosthetic joint infections in the 2018 Definition of Periprosthetic Hip and Knee Infection.

Major criteria (at least one of the following)				Decision
Two positive growth of the same organism using standard culture methods or				Infected
Sinus tract with evidence of communication to the joint or visualization of the prosthesis				
Minor Criteria	Threshold		Score	Decision
	Acute	Chronic		
Serum CRP (mg L^−1^) or	100	10	2	≥6 Infected; 3–5 Inconclusive; <3 Aseptic
D‐Dimer (ug L^−1^)	Unknown	860		
Elevated Serum ESR (mm h^−1^)	No role	30	1	
Elevated Synovial WBC (cells uL^−1^) or	10 000	3000	3	
Leukocyte Esterase or	++	++		
Positive Alpah‐defensin (signal/cutoff)	1.0	1.0		
Elevated Synovial PMN (%)	90	70	2	
Single Positive Culture			2	
Positive Histology			3	
Positive Intraoperative Purulence			3	

### 2.7 Knock‐Out the CXCR6 on Treg Can Reverse Immunosuppressive Microenvironment of PJI Model

**Figure 6:**
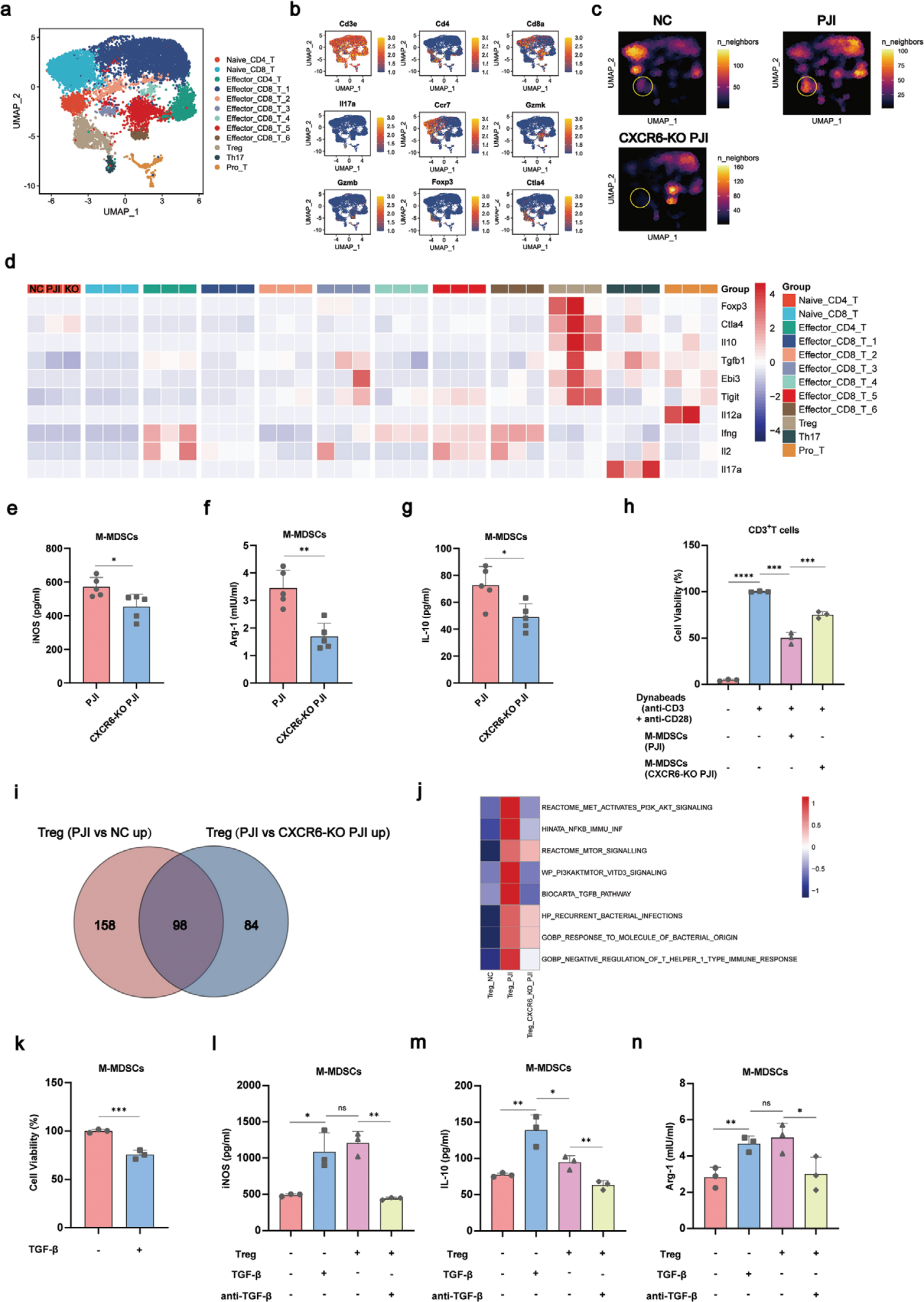
CXCR6 is a key gene for Treg recruitment in the synovial microenvironment of PJI. a) The UMAP plot showed detailed annotation of the lymphocytes subpopulations in synovial cells of mice knee joint from NC (n = 6), PJI (n = 6) and CXCR6‐KO PJI (n = 6) group. Twelve myeloid clusters were visualized. Treg Regulatory T cell, Th17 T helper cell 17, Pro_T Proliferative T cell. b) The UMAP plot showed the expression level of biomarkers for lymphocytes subpopulations. c) Changes in the composition of the lymphocytes subpopulations compartment among NC, PJI and CXCR6‐KO PJI group were visualized as cell density. d) The heatmap showed the average expression of inhibitory factors (Foxp3, Ctla4, Il10, Tgfb1, Ebi3, Tigit and Il12a) and cytokines (Ifng, Il2 and Il17a) in NC, PJI and CXCR6‐KO PJI group. e–g) Expression of iNOS, IL10 and Arg‐1 protein in M‐MDSCs between CXCR6‐KO PJI and PJI group (CXCR6‐KO PJI [flox/flox], n = 5; PJI [+/+], n = 5). Unpaired t test; *p < 0.05, **p < 0.01. h) T cell suppression assay to evaluate the capacity of M‐MDSCs to suppress T cells (n = 3). Unpaired t test; ***p < 0.001, ****p < 0.0001. i) The Venn plot illustrated the number of differentially expressed genes of Treg in the PJI group compared with the NC and CXCR6‐KO PJI groups. j) GSVA showed the upregulated pathways of Treg in the PJI group compared with the NC and CXCR6‐KO PJI groups. k) The number of synovial M‐MDSCs with or without the Treg cell‐related cytokines TGF‐β (n = 3). Unpaired t test; ***p < 0.001. l–n) IL‐10, iNOS, and Arg‐1 levels in supernatant of M‐MDSCs in different experimental groups. Anti‐TGF‐β antibody (10 mg/mL) was used to neutralize TGF‐β. Data were representative of three independent experiments (n = 3). Unpaired t test; ^ns^ *p* > 0.05, *p < 0.05, **p < 0.01.

To investigate the impact of CXCL16/CXCR6 signaling axis in *S. aureus* PJI, we established *S. aureus* PJI mouse model by implanting prosthesis and injecting *S. aureus* into the knee joint. The intra‐articular pus and prosthesis of C57BL/6J mice were showed in Figure S8a,b (Supporting Information). H&E staining of implanted knee joints of *S. aureus* PJI mice revealed that chronic infective features, including numerous necrosis, tissue inflammation and fibrotic tissue formation were obviously occurred at 35 days post‐establishment of *S. aureus* PJI mouse model. Simultaneously, Treg‐specifical CXCR6 knockout mice (CXCR6[flox/flox], FOXP3YFP‐Cre[KI/+], CXCR6‐KO PJI) were also used to establish *S. aureus* PJI mouse model (Figure S8c,d, Supporting Information). ScRNA‐seq analysis on knee synovial immune cells from negative control mice (Sham‐operated, CXCR6[+/+], FOXP3YFP‐Cre[KI/+), PJI mice (CXCR6[+/+], FOXP3YFP‐Cre[KI/+, 35d), and CXCR6‐KO PJI (35d) mice demonstrated that lymphocyte populations were classified into twelve distinct subpopulations (**Figure** **6**a,b; Figure S8f,g, Supporting Information). As to PJI mice, the amount abundant of Treg, and the anti‐inflammatory genes, including inhibitory factors IL‐10, TGFB1, and IL‐35 (EBI3‐IL12A) were significantly increased. Moreover, the anti‐bacteria functions of T cell were suppressed by the decreased expression of IL2 and IFNG on Effector_CD4_T, and IL17A on Th17 cells. Importantly, above changes were attenuated in CXCR6‐KO PJI mice (Figure 6c,d). These results suggested the infiltration of Treg were increased and the sterilizing immunity of T cells were suppressed in PJI mice, but deletion the CXCR6 of Treg could active the anti‐bacterial response of T cells by inhibiting the infiltration of Treg.

In order to further investigate the impact on M‐MDSCs following the knockout of CXCR6 on Treg, we conducted flow sorting on M‐MDSCs from the PJI mice and CXCR6‐KO PJI mice. Compared to M‐MDSC from PJI mice, the M‐MDSCs from CXCR6‐KO PJI mice displayed significant reductions of IL10, iNOS and Arg‐1 which were involved in MDSCs‐induced immunosuppressive activity (Figure 6e–g), and they also lost the ability to suppress T cell proliferation (Figure 6h). These results suggested that under the *S. aureus* biofilm milieu of PJI, immunosuppressive functions of M‐MDSCs were abolished as CXCR6 of Treg was deleted, implying a regulatory role of Treg on M‐MDSCs.

Next, to study the regulatory mechanism of Treg on M‐MDSCs, differential analysis was conducted on the Treg of the NC group, PJI group and CXCR6‐KO PJI group, respectively. Venn plot showed the screening of 256 (PJI versus NC) and 182 (PJI versus CXCR6‐KO PJI) increased DEGs, followed by the screening of 98 crossover (Figure 6i). The GSVA enrichment analysis revealed that the TGFB pathway was significantly more enriched in the PJI group compared to both the NC group and CXCR6‐KO PJI group (Figure 6j). TGFB1 was also present in the 98 crossover upregulated DEGs. Lee's research showed that TGF‐β was a crucial controller of MDSCs proliferation and functions.^[^ ^42^ ^]^ Therefore, Treg possibly regulate the immunosuppressive functions of M‐MDSCs by secreting TGF‐β. To confirm this important role of TGF‐β in regulating M‐MDSCs, we cultured bone marrow of mice and induced them to differentiate into M‐MDSCs. Addition of TGF‐β directly to M‐MDSCs caused the number of M‐MDSCs to increase (Figure 6k). Then, we cultured M‐MDSCs with Treg sorted from PJI mice, TGF‐β, and anti‐TGF‐β, respectively. TGF‐β/Treg significantly enhanced the production of IL10, iNOS and Arg‐1 of M‐MDSCs. Meanwhile, anti‐TGF‐β could reduce the secretion of the aforementioned factors (Figure 6l–n). These results indicated that TGF‐β could active the proliferation and immunosuppressive functions of M‐MDSCs. In summary, the above findings suggested that Treg were raised into *S. aureus* infected synovium by expression of CXCR6, furthermore, the infiltrated Treg triggered the immunosuppressive environment not only by suppressing T cell anti‐bacterial functions, but also by activating M‐MDSCs.

### 2.8 Verify the Efficiency of CXCR6 as a Therapeutic Target of PJI

**Figure 7:**
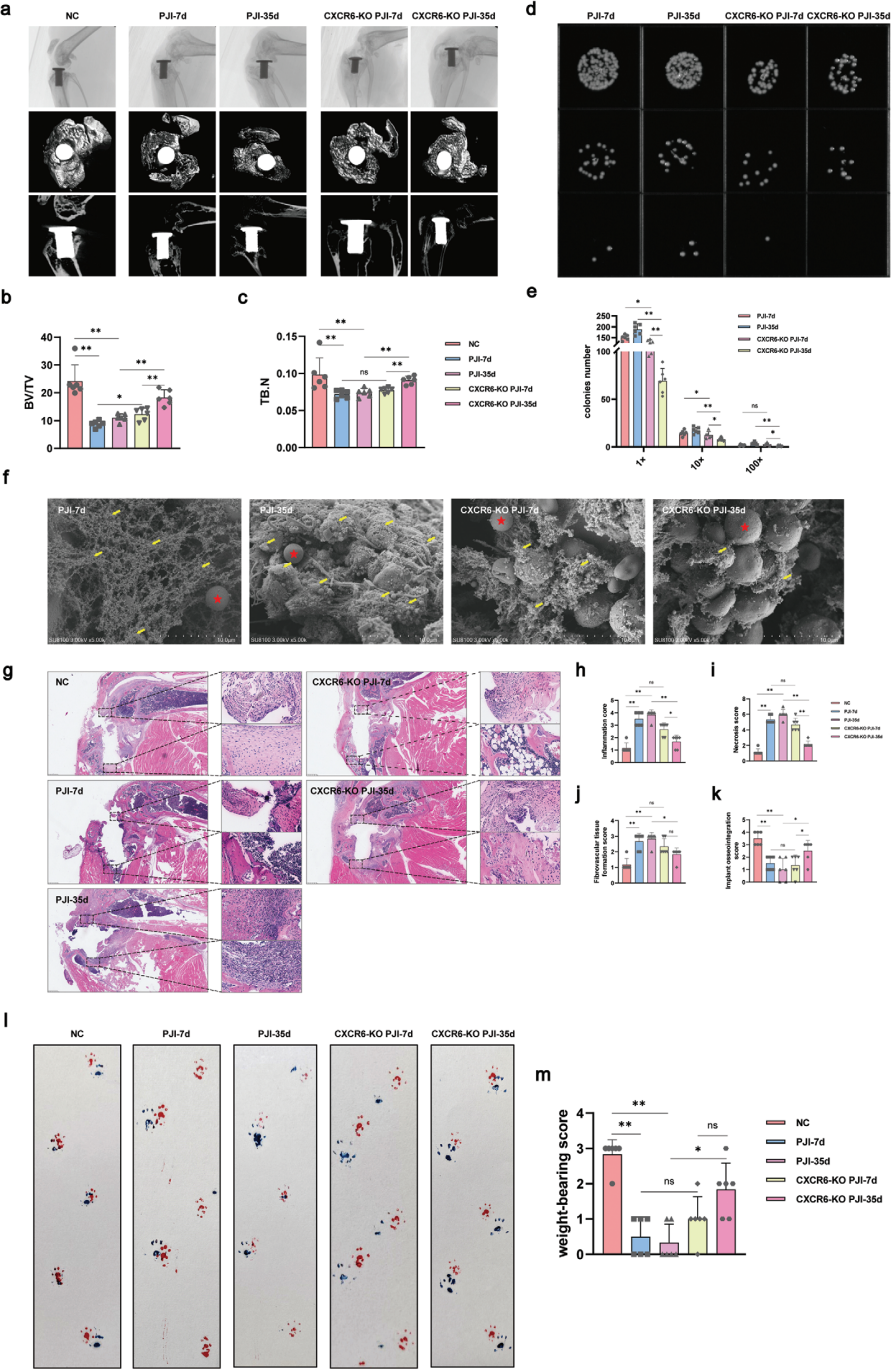
Target CXCR6 inhibit PJI progression in Treg CXCR6 specific KO PJI mouse model. a) Representative sagittal X‐ray images, 3D reconstruction images of horizontal views and sagittal views demonstrating knee synovial tissues and bones changes from NC, PJI, and CXCR6‐KO group at different time points (7day, 35day) (n = 6, per group). b,c) Quantitative micro‐CT analysis of bone volume fraction (BV/TV) and Trabecular Number (TB.N). Unpaired t test; ^ns^ *p* > 0.05, *p < 0.05, **p < 0.01. d) The representative images depicted colony forming units (CFUs) of *S. aureus* on the surface of the prosthesis, which were diluted 10‐fold (10X) and 100‐fold (100X) in the original solution (1X). (n = 6, per group). e) The numbers of bacteria isolated from the surface of the prosthesis. Unpaired t test; ^ns^ *p* > 0.05, *p < 0.05, **p < 0.01. f) The formation of *S. aureus* biofilm on the surface of prosthesis under SEM. The red star represented Ti particles; The yellow arrow represented *S. aureus* biofilm. g) Representative H&E‐stained photomicrographs of knee joint sagittal sections (n = 6, per group). h–k) Quantitative analysis of histopathological scoring including necrosis, inflammation, fibrovascular tissue formation and implant osseointegration score. Unpaired t test; ^ns^ *p* > 0.05, *p < 0.05, **p < 0.01. l) Weight‐bearing was assessed to demonstrate the motor function of mice (n = 6, per group). Forelimbs (dark blue ink); hindlimbs (red ink). m) The grade of weight‐bearing activity among different groups. Unpaired t test; ^ns^ *p* > 0.05, *p < 0.05, **p < 0.01.

To examine the effect of CXCR6 deletion on Treg for the treatment of *S. aureus* PJI, we evaluated the elimination of *S. aureus* and biofilm, and the knee‐joint function of PJI mice and CXCR6‐KO PJI mice at two time points (7day, 35day). First, the radiographs showed that osteolytic resorption, enlargement of the bone tunnels, destruction and thinning of the trabecular microstructure, and thinning of the bone cortex became more and more serious from 7d to 35d post infection. These injuries at 7d in CXCR6‐KO PJI mice were not as terrible as that at 7d in PJI mice. Surprisingly, with the time increased, these injuries nearly recovered at 35d in CXCR6‐KO PJI mice. BV/TV and TB.N values showed the similar tendency in all groups. Second, *S. aureus* burden (**Figure** **7**d,e) and biofilm formation (Figure 7f) surrounding the prostheses were assessed at 7d and 35d post infection in both PJI mice and CXCR6‐KO PJI mice. Interestingly, as time increased, the *S. aureus* burden and biofilm formation were increased in the PJI group, but both of them decreased in the CXCR6‐KO PJI group. Especially, at the 35d of CXCR6‐KO PJI mice, most biofilm were eliminated. Furthermore, according to the histological analysis (Figure 7g) of implanted knee joints (including tibia and femur), the amounts of tissue necrosis, inflammation, implant osseointegration and fibrotic tissue formation were scored to define the degree of infection. There was no statistically significant difference in the score between the PJI group and the CXCR6‐KO PJI group at 7d. However, the CXCR6 KO PJI group showed a decrease in inflammation, necrosis and fibrotic tissue formation scores, and an increase in implant osseointegration score compared to the PJI group at 35d (Figure 7h–k). Above results indicated knocking out CXCR6 from Treg resulted in a notable increase in getting rid of biofilm and bacterial. Moreover, it effectively reduced the histological damages caused by bacterial infection. For joint motor function, our findings revealed that weight‐bearing activity of mice was significantly decreased following PJI, while knocking out CXCR6 from Treg improved weight‐bearing activity to a level similar to that of the NC group at 35d (Figure 7l,m). In summary, knockout of CXCR6 from Treg could effectively repair the tissue around knee joint and restore the function of knee joint by reducing the *S. aureus* infection and biofilm formation.

## 3 Discussion

The selective targeting of Treg presents an appealing strategy for overcoming the immune‐suppressive nature, liberating immune effector cells to eradicate bacteria. Glaubitz's study demonstrated that Treg could serve as a promising therapeutic target for preventing bacterial infections during severe acute pancreatitis, thus improving the disease course and outcomes.^[^ ^75^ ^]^ The administration of Compound 511 mitigated lung injuries induced by methicillin‐resistant *Staphylococcus aureus* (MRSA) through the inhibition of Treg‐mediated immunosuppression in mouse model.^[^ ^76^ ^]^ Administration of citrulline effectively reduced the expansion of sepsis‐induced Treg and MDSCs, which inhibited the progression of secondary pneumonia caused by MRSA.^[^ ^39^ ^]^ The application of Treg as a therapeutic target in *S. aureus* PJI has not yet been previously explored. Our study reveals that the synovium of *S. aureus* PJI exhibits an immunosuppressive microenvironment characterized by increased quantity and activity of M‐MDSCs and Treg. Mechanistically, *S. aureus* biofilm induces the production of CXCL16 from M‐MDSCs, which recruit Treg by the specific receptor CXCR6. In turn, increased Treg secretes TGF‐β to enhance the proliferation and immunosuppressive function of M‐MDSCs. This positive feedback loop between Treg and M‐MDSCs leads to a cascade reaction that suppresses the sterilizing immunity against *S. aureus* in PJI. Importantly, Treg‐specific CXCR6 knockout effectively reverses the immunosuppressive microenvironment, subsequently leading to the suppression of *S. aureus* growth and biofilm formation. Ultimately, the infection and functional impairment of the knee joint are alleviated.

Implant‐infecting bacteria are able to evade both biocides and antibiotic therapy.^[^ ^14^ ^]^ The application of antibiotics is integral to the entire process of PJI treatment, however, drug resistance due to prolonged infection is inevitable. The resistance rate of erythromycin and clindamycin in treating PJI caused by *S. aureus* was ≈50%, while penicillin even exceed 80%.^[^ ^77^ ^]^ Bacterial attachment to the surrounding prosthesis facilitates biofilm formation, further allowing for high tolerance to antibiotics.^[^ ^78^ ^]^ The interaction of antimicrobials with biofilm matrix components prevents bacteria from being eliminated.^[^ ^79^ ^]^ Furthermore, the biofilm formation generated a niche of immune depression, including invalidity of T cell priming, exhaustion of antigen‐presenting cells (APCs) and waning of antibody responses.^[^ ^80^ ^]^ Recently, many studies have suggested that immunotherapy represents a promising novel approach for infectious diseases.^[^ ^81^, ^82^, ^83^ ^]^

Our study shows the quantity and immunosuppressive function of both M‐MDSCs and Treg are increased in response to *S. aureus* biofilm depending on CXCL16/CXCR6 receptor‐ligand pair, which has implicated in tumorigenesis, autoimmune disorders and infectious diseases.^[^ ^47^, ^84^, ^85^ ^]^ Target of this CXCL16/CXCR6 has been extensively investigated to switch immune cells into either an inhibitory or activating state.^[^ ^44^, ^45^ ^]^ Wu's study indicated that the activation of the CXCL16/CXCR6 exacerbated keratitis by increasing corneal bacterial load.^[^ ^46^ ^]^ Ashhurst's study showed that the deficiency of CXCR6 in mice resulted in inhibition of bacterial and viral infections.^[^ ^43^ ^]^ Therefore, CXCL16/CXCR6 has been reported to be involved in the suppression of bactericidal immunity in many infectious diseases. Actually, chemokines and their receptors has been extensive applied as therapeutical target, primarily focusing on tumor immunotherapy.^[^ ^86^, ^87^ ^]^ The inhibition of CXCR2 in pancreatic tumors prevented metastatic spreading by enhancing the T cell response and improving sensitivity to anti‐PD‐1 therapy.^[^ ^88^, ^89^ ^]^ This is also supported by our finding that targeting the CXCR6 in Treg, the specific receptor of CXCL16, can effectively prevent the progression of *S. aureus* PJI. Although both CXCL16 and CXCR6 can be served as targets for disrupting the interplay between M‐MDSCs and Treg, the expression of CXCL16 has been observed more than in M‐MDSCs, also including monocytes, macrophages and dendritic cells. Therefore, deletion of CXCL16 possibly affect the functions of other immune cells. Norimitsu's study suggested that CXCL16 plays a crucial role in both phagocytosis of bacterial antigens and T helper 1 immune response. Its absence leads to undermine immune response against Bacteroidetes.^[^ ^90^ ^]^ Consequently, CXCL16 is not as viable target as CXCR6 for *S. aureus* PJI. Indeed, the inhibition of CXCR6 can progressively better the infection of PJI over time, indicating the reactivation of antibacterial immunity. As to antibiotics, it typically exerts their antibacterial function in the early stages of PJI; however, their effects are gradually diminished with the time increasing due to antimicrobial resistance. This further confirmed the important role of immunotherapy of CXCR6 in response to *S. aureus* PJI.

Growing evidence from various study demonstrates that CXCR6‐deficency mice is a practical, successful and sustainable animal model for the establishment of different pathogen models (such as lung infection, tumor, IBD, inflammatory arthritis) in different time points (from 7d to 80d).^[^ ^43^, ^91^, ^92^, ^93^, ^94^ ^]^ As to these studies, the CXCR6‐deficient mice seldom exhibited impairments or significant adverse phenotypes under standard protocol.

CXCR6+ T cells have also been involved in many immune‐related diseases, such as lung infection, inflammatory arthritis, psoriasis, multiple sclerosis and Crohn's disease.^[^ ^43^, ^51^, ^94^, ^95^, ^96^ ^]^ It's reported that CXCR6‐deficient mice (6 weeks) demonstrated enhanced host control over Mycobacterium tuberculosis and influenza virus by influencing the kinetics of the inflammatory response.^[^ ^43^ ^]^ In CXCR6‐deficient mice (80 days), T cells showed impaired cytokine polarization, leading to fewer IL‐17A‐ and IFNγ‐producing cells. This reduction contributed to a lower incidence of arthritis, milder disease severity, and less T cell accumulation.[^94^ ^]^ These findings position CXCR6 as a potential therapeutic target for managing immune‐related diseases.

So far, given the specific function of CXCR6+ T cells and their small proportion within peripheral immune organs and peripheral blood, there have been no reports indicating that targeting them with an anti‐CXCR6 mouse antibody or through CXCR6 KO would result in severe systemic side effects.^[^ ^47^, ^92^ ^]^ Although CXCR6 deficiency may lead to defective T cell migration and retention in peripheral tissues,^[^ ^91^, ^97^, ^98^ ^]^ the functional relevance of CXCR6 in T cell biology within the PJI microenvironment remains unknown.

Treg induces immunosuppression through diverse mechanisms. The expression of CD80/CD86 costimulatory molecules on APCs could be down‐regulated by CTLA‐4 on Treg, resulting in dysfunction of dendritic cells and rendering them inadequate for combating *S. aureus* infection.^[^ ^99^, ^100^ ^]^ In addition, Treg secrete cytokines to remodel immunosuppressive microenvironment, such as IL‐10, TGF‐β, and IL35. Detailed, IL‐10 has been reported to facilitate the formation of bacterial biofilm, thereby enabling *S. aureus* to escape from the host immune clearance.^[^ ^101^, ^102^, ^103^ ^]^ IL35 could suppress the differentiation of CD4+T cells into Th17 cells, thereby dampening the immune response against infections.^[^ ^104^ ^]^ TGF‐β suppressed the immune activities of Th1 and Th2 cell.^[^ ^105^ ^]^ Our scRNA‐seq studies revealed a significant enrichment of genes involved in above cytokines in Treg from *S. aureus* PJI, meaning that the anti‐bacterial activity of host would be suppressed by the Treg. Of note, Treg‐secreted TGF‐β also has been reported to up‐regulate the immunosuppressive function and proliferation of M‐MDSCs.^[^ ^59^ ^]^ Wang's study showed that TGF‐β induced the expansion and functional reprogramming of MDSCs via the TGF‐β/Smad pathway.^[^ ^106^ ^]^ Zeng's study also demonstrated the proliferation of MDSCs could be induced by enhancing translation of TGF‐β.^[^ ^107^ ^]^ After the specifical deletion of CXCR6 in Treg, the immunosuppressive function and proliferation of M‐MDSCs also decreased, reinforcing the interplay between Treg and M‐MDSCs. Based on the increasing expression of TGF‐β in Treg, coupled with the observation that Treg is critical for improving M‐MDSCs immunosuppressive activity and proliferation through producing TGF‐β in our study.

The potential applications of CXCR6 may extend beyond PJI. All types of implants in the human body carry a risk of infection, closely linked to biofilm formation resulting from bacterial colonization. The presence of biofilm on silicone hydrogel contact lenses can lead to keratitis in compromised corneas, while a compromised host immune response hinders the clearance of infection.^[^ ^108^ ^]^ Following interventional implantation of heart valves, biofilms may develop on the implants, resulting in severe infective endocarditis, which is closely associated with the development of immunosuppression.^[^ ^109^ ^]^ Our target has wide potential applicability for multiple implant‐related infections.

There are several limitations to our study. First, *S. aureus* is the only strain used in this study; further research should investigate whether the CXCL16‐CXCR6 axis has similar effects on different or multiple microbes and how this axis functions in interactions between microbes and immune cells within the PJI microenvironment. Second, since there was no specific CXCR6+ Treg‐targeted monoclonal antibody available for our study, it is necessary to develop such a specific monoclonal antibody and evaluate potential adverse events in both the short and long term, including immune‐related toxicities. Emphasis should also be placed on dose optimization, monitoring, and cost‐effectiveness in future clinical applications. Finally, further experiments, including cytokine measurements (ELISA/Multiplex Assays), immune cell profiling (flow cytometry/IHC), gene expression profiling, Delayed‐Type Hypersensitivity assays, and monitoring for Cytokine Release Syndrome, need to be designed to assess safety concerns, such as the risk of immune suppression or cytokine‐related syndromes associated with immunotherapy strategies.

The infection‐related interplay observed in both M‐MDSCs and Treg following *S. aureus* PJI showed in this study, may present a pivotal part of host's anti‐bacterial response. Therefore, the innovative advantage of this study lies in the identification of a robust regulator CXCR6 that effectively enhances the immunosuppressive activity of Treg and M‐MDSCs in *S. aureus* PJI. Although targeting CXCR6 alone is not sufficient to clear the infection, it significantly impedes the progression of *S. aureus* PJI. Therefore, our study indicates that immunotherapy is a promising therapeutic option for *S. aureus* PJI. In the future, a combinational approach that combines the blockage of CXCR6 with conventional antibiotics is expected to be more effective in combating PJI.

## 4 Experimental Section

All research activities were pre‐approved by The Institutional Research Ethics Committee of The First Affiliated Hospital, School of Medicine, Sun Yat‐sen University ([2021]676), and Informed consent were obtained for all human participants.

### Single‐Cell RNA Sequencing

*Preparation of single cell suspensions*: The fresh tissues obtained from patients with PJI, AF and OA were enzymatically digested to obtain a single‐cell suspension. The synovial tissues obtained during the operation was segmented into small pieces (1 cm^3^) with a scalpel, and then promptly immersed in the cell preservation solution (Absin, China). After removing visible fat and connective tissues, the samples were washed with cold PBS at 4 °C. Subsequently, the tissues were minced and digested using a dissociation enzyme prepared with type I collagenase (200 u ml^−1^) and hyaluronidase (50 u ml^−1^), followed by incubation at 37 °C for 10–15 min. The resulting cell suspension was filtered through magnet‐activated cell sorting SmartStrainer (70 µm) (Miltenyi Biotec). Following centrifugation of the cell suspension at ≈1500 rpm for 5 min, supernatant was discarded. The cells were then washed twice with PBS to determine their viability, which was maintained above 90%.

### scRNA‐Seq Data Processing

10x GemCode Technology was utilized to individually index the transcriptome of each cell. Prepared cells were transformed into barcode scRNA‐seq libraries. The resulting scRNA‐seq data were aligned against the reference genomes GRCh38 and GRCm39 for human and mouse samples, respectively, using CellRanger (version 5.0.1) to generate a raw gene expression matrix. Cells with low quality were eliminated based on two criteria: 1) the number of detected genes <200; 2) the percentage of mitochondrial genes exceeding 25%. Next, it was used DoubletFinder (2.0.3)^[^ ^110^ ^]^ to remove potential doublet cells. Following rigorous quality control measures, a total of 134 756 cells from 13 human samples (6 PJI, 3 AF and 3 OA), as well as 19 712 cells from 18 mouse samples (6 NC, 6 PJI and 6 CXCR6‐KO PJI) were retained.

### Unsupervised Clustering and Annotation

First, the “NormalizeData” function to homogenize the data with the scaling parameter of 10 000 was used, followed by log‐transforming the data. The “FindVariableGenes” function was used to identify 2000 highly variable genes with selection method of “vst”. Subsequently, the data was standardized by “ScaleData” function. For cluster analysis, Principal Component Analysis was performed with dimensions set at 20 and a resolution of 0.5. Initially, seven major cell types based on known characteristic markers was defined, Fibroblasts (THY1, PRG4, and COL1A1), Lymphocytes (CD4, CD3D, and CD3E), Myeloid cells (CD163, CD68, and LYZ), Endothelial cells: (VWF and PECAM1), Mural cells (ACTA2), Mast cells (TPSAB1 and TPSAB2), Cycling cells (TOP2A and MKI67). It was then repeated these operations to define more detailed cell subpopulations. To correct for batch effects resulting from differences in sample collection time points, we employed Harmony^[^ ^111^ ^]^ package (version 0.1.0). Subsequently, the “FindMarkers” function was used to identify characteristic genes specific to each cluster relative to others. The differential expression with >0.25‐fold difference and P value <0.05 with Wilcoxon test were considered significant. Visualization of gene expression levels was achieved through VlnPlots, DotPlots and FeaturePlots.

### Differential Gene Expression and Enrichment Analysis

The analysis of DEGs among three groups, including PJI, AF, and OA groups, was calculated using the “FindMarkers” function in the Seurat package, based on the Wilcoxon Rank‐Sum test. DEGs were inputted into ClusterProfiler package to identify enriched functional annotations. Gene set collections from databases such as Gene Ontology and Kyoto Encyclopedia of Genes and Genomes was utilized. The resulting differential enrichment pathways were visualized through bubble plots and heatmaps.

### Enrichment Analysis of Gene Sets Based on GSVA

The scRNA data that had been normalized and annotated was prepared. The GSVA package^[^ ^112^ ^]^ (v 1.38.2) to perform gene set enrichment analysis on scRNA data was applied, aiming to assess the activity or enrichment of predefined gene sets within each cell subpopulation. The pathways/gene sets were derived from the MsigDB database (https://www.gsea‐msigdb.org/gsea/msigdb/index.jsp). After obtaining the matrix of GSVA scores, a differential analysis of pathways or gene sets using the limma package (v 3.46.0) was performed. A significance level of P < 0.05 was considered statistically significant. The results were visualized through heatmaps.

### Developmental Trajectory Analysis

To establish the developmental trajectory of T cells, myeloid cells, endothelial cells, and fibroblasts, the Monocle 3 package^[^ ^113^ ^]^ based on the gene expression matrix was employed. Initially, the “preprocess_cds” function to compute a low‐dimensional space that would serve as the input for subsequent dimensionality reduction was applied. Subsequently, the “reduce_dimension” function was used to perform dimensionality reduction on the data. Following this step, the “Embedding” function to obtain cell coordinates or feature expressions in the low‐dimensional space was used. Finally, the “learn_graph” and “order_cells” functions to construct the pseudotime trajectory accurately was utilized.

### Scoring of Cell Types and Signature

The “AddModuleScore” function in the Seurat package was utilized for scoring the gene expression signatures of myeloid cells and T cell subpopulations. Functional genes were analyzed, encompassing naive score, exhausted score, regulatory score, cytotoxic score of T cells,^[^ ^114^ ^]^ and MDSCs score of myeloid cells.^[^ ^62^ ^]^

### Single‐Cell Data from Databases

The single‐cell data gene expression matrices of single‐cell data were obtained from GEO and the ImmPort databases (https://www.immport.org/shared/study). All selected datasets consisted of gene expression data derived from synovial tissues affected by OA or RA. Dataset SDY998, comprising 4 OA and 20 RA samples was sourced from ImmPort database.^[^ ^115^ ^]^ Dataset GSE202375, which included 5 RA samples, underwent sequencing on both the Illumina HiSeq 2500 and Illumina NextSeq 500 platforms.^[^ ^116^ ^]^ Dataset GSE152805, encompassing 3 OA samples, was sequenced using the Illumina HiSeq 4000 platform.^[^ ^117^ ^]^ These two datasets were retrieved from the GEO database.

### Cell‐Cell Interactions

Cellchat^[^ ^118^ ^]^ to investigate intercellular communication among distinct cell types in the synovium tissues of the knee joint, specifically focusing on PJI, AF, and OA was utilized. The number of interactions and interaction weights/strength between cell types were evaluated by examining ligand and receptor expression levels within each cell type. Afterward, it was identified potential ligand‐receptor pairs responsible for mediating this intercellular communication and compared them across groups. The cell‐cell communication networks were visualized using dot plots, circle plots or heatmaps, emphasizing significant interactions between different cell types.

### Milo Analysis of Differential Abundance

The miloR^[^ ^119^ ^]^ package offers modular functions for conducting differential abundance testing on replicated single‐cell experiments. The single‐cell data to incorporate information about neighborhoods on the KNN graph and integrated into the Milo object was expanded. Following the definition of representative neighborhoods on the KNN graph, miloR to exploit variations in cell numbers between replicates under the same experimental condition, enabling us to test for differential abundance was utilized. The results were visualized through an abstracted graph of neighborhoods, where each node represented a neighborhood and edges indicated shared cell counts between two neighborhoods. The arrangement of nodes was determined by the position of the index cell in the UMAP embedding of all single cells. Neighborhoods exhibiting significant cell subpopulations were color‐coded based on their log‐fold change values. Furthermore, a cell type label to each neighborhood by identifying the most abundant cell type within its constituent cells was assigned. The beeswarm plot was employed to visualize differential abundance across different cell types.

### Bulk‐Seq Analysis

To further validate the findings from scRNA‐seq, we employed bulk RNA‐seq, which provides a snapshot of gene expression patterns by quantifying the abundance of RNA transcripts. Bulk sequencing was performed on samples of PJI and AF synovial tissues (PJI:AF = 18:18). Fresh synovial tissue was obtained from patients during surgery, treated with RNA stabilization solution and stored at −80 °C for RNA extraction. Total RNA was extracted using RNAiso Plus reagent (Takara, 9108, Japan) according to the manufacturer's instructions. Synovial RNA samples were sent to Gene Denovo Biotechnology in Guangzhou, China for library creation and sequencing. RNA quality assays were evaluated using the Agilent 2100 Bioanalyzer (Agilent RNA 6000 Nano Kit). Total RNA was sequenced from RNA samples with stranded and 150 BP paired‐end reads using an Illumina Novaseq 6000 system. 28 immune cell types and their featured gene profiles were obtained from the study by reference to Charoentong et al. The ssGSEA (single‐sample Gene Set Enrichment Analysis)^[^ ^120^ ^]^ of the GSVA R package and estimate R package were used to analyze the immune cell infiltration of each cell type and to scale the enrichment scores to indicate the relative level of infiltration. Immune function and immunosuppression were calculated based on the enrichment scores of the infiltrating cell types for the cell type that performs the immune function (activated CD4 T cell, activated CD8 T cell, central memory CD4 T cell, central memory CD8 T cell, effector memory CD4 T cell, effector memory CD8 T cell, type 1 T helper cell, type 17 T helper cell, activated dendritic cell, CD56 bright natural killer cell, natural killer cell, and natural killer T cell) and the cell type that performs the immune suppression (regulatory T cell, type2 T helper cell, CD56 dim natural killer cell, immature dendritic cell, macrophage, MDSC, neutrophil, plasmacytoid dendritic cell).^[^ ^121^ ^]^

### Immunofluorescence

Formalin‐fixed paraffin‐embedded blocks of PJI, AF, and OA samples of knee joint synovial tissues were sectioned into slides. The tissue sections underwent dewaxing using xylene, hydration, and antigen retrieval. Subsequently, they were blocked with 10% goat serum at room temperature for 30 min. Following this, the primary antibody was applied to stain the tissues, followed by incubation with a horseradish peroxidase‐conjugated secondary antibody (Goat Anti‐rabbit IgG). Tyramide signal amplification substrates were then used for visualization. Immunofluorescence staining was performed after elution of the antigen‐antibody complexes. The steps involved in immunohistochemistry were similar to those in immunofluorescence. The primary antibodies were used in immunofluorescence: anti‐CXCL16 (Abcam, ab273116, 1:500), anti‐CXCR6 (Affinity Biosciences, DF2328, 1:500), anti‐CD68 (Abcam, Cat# ab129024, 1:500), anti‐FOXP3 (Abcam, Cat# ab129024, 1:500), anti‐CD4 (Abcam, Cat# ab129024, 1:500), anti‐S100A8 (Proteintch, 15792‐1‐AP, 1:500), anti‐S100A9 (Proteintch, 26992‐1‐AP, 1:500).

### Flow Cytometry and Sorting

The CYTEK Aurora flow cytometer was utilized for flow cytometry analysis. Cell suspensions were prepared and stained with Fixable Viability Stain 700 (BD Biosciences, diluted at 1:1000, catalog number 564 997) at a temperature of 4 °C in the absence of light for 20 min. Subsequently, the cells were incubated with Human Fc block (BD Pharmingen, catalog number 564 219) at a temperature of 4 °C in the dark for 15 min. For extracellular staining, fluorochrome‐labeled antibodies were added to the cells and incubated at a temperature of 37 °C for 20 min. The cells were fixed and permeabilized with Fix/Perm buffer (BD Biosciences, 562 574) at 4 °C for 40 min for intracellular FOXP3 staining, following the manufacturer's recommendations. FlowJo software (Treestar, v10.8.1) was employed for data analysis. Flow sorting procedures were conducted using the BD FACSAria III cell sorter equipped with a nozzle size measuring 70 mm in diameter. Live myeloid cells (CD45+CD11b+) and T cells (CD45+CD3e+) were performed on the BD FACSAria III instrument. The primary antibodies were used in flow cytometry and sorting: anti‐human CD45 (BD Biosciences, BUV395, 563 792), anti‐human CD3 (BD Biosciences, BV786, 563 800), anti‐human CD4 (BD Biosciences, BV510, 562 970), anti‐human FOXP3 (ThermoFisher, 17‐4776‐41), anti‐human CD25 (BD Biosciences, PE, 555 432), anti‐CD11b (BD Biosciences, FITC, 557 396), anti‐human CD33 (BD Biosciences, BV421, 562 854), anti‐human HLA‐DR (BD Biosciences, APC‐H7, 561 358), anti‐human CD14 (BD Biosciences, PE‐Cy7, 562 698), anti‐human CD15 (BD Biosciences, PerCP‐Cy5.5, 560 828), anti‐mouse CD45 (BD Biosciences, PE, 553 081), anti‐mouse CD3e (BD Biosciences, APC, 553 066), anti‐mouse Ly6C (BD Biosciences, PE‐Cy7, 560 593), anti‐ mouse Ly6G (BD Biosciences, APC, 560 599), anti‐mouse Foxp3 (BD Biosciences, Alexa Fluor 647, 560 402)

### ELISA

M‐MDSCs were isolated from mouse bone marrow via the Myeloid‐Derived Suppressor Cell Isolation Kit (Miltenyi Biotec) according to the manufacturer’ s protocol. Supernatants were collected to determine the IL‐10, Arg‐1, and iNOS concentration of cell culture supernatants. The above indicators were determined in M‐MDSCs, as well as in groups and after the addition of Treg, TGF‐β, and anti‐TGF‐β. The contents of iNOS (EM0272) and Arg‐1 (EM0525) were obtained from FineTest (Wuhan, China). The contents of IL‐10 (70‐EK210/4‐96) was obtained from MultiSciences (Hangzhou, China). CXCL16 in human synovial fluid samples was also measured by ELISA (70‐EK1254‐96, Hangzhou, China, MultiSciences). The measurements were conducted following the instructions.

### Cell Counting kit‐8 (CCK‐8) Assay

CD3^+^ T cells (1.5 × 10^5^ cells well^−1^) were isolated from the synovium and cultured in 96‐well plates with Dynabeads (Invitrogen) coated with anti‐CD3 and anti‐CD28 antibodies. M‐MDSCs were isolated via FACSAria III flow cytometer (BD Biosciences) on the basis of cell surface marker staining and isolated cells (3 × 10^5^ cells well^−1^) were co‐cultured with CD3^+^ T cells. Then, the viability of CD3^+^ T cells was evaluated. Besides, the viability of M‐MDSCs was evaluated with or without the addition of TGF‐β.

Viability of T cells and M‐MDSCs were evaluated using a CCK‐8 assay kit (Huayun Biotechnology Co., Ltd., Guangzhou, China). The cells were seeded into 96‐well plates (2  × 10^3^ cells well^−1^) and cultured for 48 h. Then, 10 µL of the CCK‐8 solution was added to each well and incubated at 37 °C for 4 h. Absorbance was measured at 450 nm using a microplate reader (Bio‐Rad iMark). Each group was set up in three wells, and the experiment was independently repeated three times.

### PJI Model

C57BL/6 mice were obtained from Jicui company (Jiangsu, China), while CXCR6 conditioned knockout (CKO) mice (CXCR6[flox/flox], FOXP3YFP‐Cre[KI/+]) and corresponding WT mice were acquired from Saiye company (Nanjing, China). The mice were housed in a specific‐pathogen‐free animal facility and provided with ad libitum access to food and water. A total of 64 male C57BL/6 mice aged 8–12 weeks were included in the experiments. Under general anesthesia, an 8 mm para‐patellar incision was made on the right knee of the mice. The longitudinal fibers of the quadriceps mechanism were separated medially, and the patella was laterally dislocated to expose the tibial plateau. During surgery, a surgical microscope was used to remove both the anterior cruciate ligament and meniscus. A fine drill bit (0.5 mm in diameter) was then used to remove the articular cartilage and proximal metaphysis up to the insertion level of the posterior cruciate ligament for implant accommodation. Subsequently, a hole with a diameter of 0.9 mm was drilled into the medullary canal. The 3D‐printed titanium implant was carefully pressed into this hole until its top aligned with that of the proximal part of tibia using a compression technique to prevent early micromotion and minimize interface gaps. Prior to closing wound, full‐range motion confirmation for knee joint took place. In addition, ≈10^4 bacteria‐forming units (CFUs) of *S. aureus* suspended in 2 µL solution were injected into the articular region for PJI group.^[^ ^122^ ^]^

### *S.aureus* Biofilm Growth

Biofilm was cultured using *S. aureus* strain USA300. Bacteria were grown on fresh plasma agar plates in glycerol stock solution. A single colony was inoculated in 25 mL of RPMI‐1640 medium added with 10% FBS, 1% HEPES and 2 ml of L‐glutamine and incubated at 37 °C, 250 rpm for 16h. As previously described, the culture was inoculated into 96‐well plates coated with 20% human plasma after 1:100 dilution (OD600 of 0.05). Biofilm was statically incubated at 37 °C, with approximately half of the medium replenished every 24 h, taking care to protect the biofilm structure.

### MicroCT Analysis

The knee joint was fixed in 4% paraformaldehyde for 24 h. High‐resolution micro‐CT (BRUKER, USA) was utilized to scan the mouse knee joint, and all images were acquired with consistent parameters (Source Voltage = 70 kV, Source Current = 200 µA, Filter = Al 0.5 mm, Scaled Image Pixel Size = 10 µm). Three‐dimensional reconstruction of the knee joint bones was conducted by contouring transverse/longitudinal image slices within a uniformly defined region of interest. The image data obtained from these knee joints were employed to reconstruct and quantify cortical bone destruction as well as associated changes in bone mass surrounding the site where the prosthetic implantation occurred. Trabecular bone measurements were performed in the area of prosthesis implantation at the distal tibia to evaluate microstructural parameters including BV score (BV/TV) and trabecular bone number (Tb. N).

### Quantification of Bacteria Adherent to the Implants and Knees

By day 35 post‐infection, mice were euthanized for the retrieval of implants and hindlimbs. In order to assess bacterial burden in the peri‐prosthetic joint, 200 ul of exudate was dissected from the *S. aureus*‐infected hindlimb and subsequently homogenized in 800ul of PBS. The homogenates were vortexed and serially diluted in PBS (10x, 100x). Each sample was cultured on TSB agar plates at 37 °C for 12 h, followed by enumeration of bacterial colonies.

### SEM Analysis

Take out the knee prosthesis, gently rinse with PBS to remove blood stains on the surface of the sample, protect the surface to be scanned and mark it (such as cutting corners on the opposite side). Quickly put into the electron microscope fixative and fix at room temperature for 2 h, then transfer to 4 °C for storage. Pay attention to protect the scanning surface to avoid violent shaking. Rinse the fixed samples 3 times (15 min time^−1^) with 0.1 M phosphate buffer PB (PH7.4). Prepare 1% osmic acid (OsO4) in 0.1 M phosphate buffer PB (PH7.4) and fix at room temperature in the dark for 1–2 h. Rinse 3 times with 0.1 M phosphate buffer PB (PH7.4), 15 min each time. Dehydrate the sample with 30%‐50%‐70%‐80%‐90%‐95%‐100%‐100% alcohol and isoamyl acetate in sequence, 15 min each time. After dehydration, the samples were dried using a critical point dryer. Place the sample tightly on the conductive carbon film double‐sided tape and place it on the sample stage of the ion sputtering instrument for conductive treatment of the gold‐sprayed sample. After the sample processing was completed, the scanning electron microscope (HITACHI, Japan) was photographed under unified parameter settings.

### Evaluation on the Weight‐Bearing Activities of Mice

The weight‐bearing activity of mice was evaluated using ink blot analysis and graded for each mouse as full (3 points), partial (2 points), toe‐touch (1 point), or non‐weight‐bearing (0 points).^[^ ^123^ ^]^ The forelimbs of the mice were coated with dark blue ink, while the hindlimbs were coated with red ink.

### Quantification and Statistical Analysis

Sequencing data were analyzed and visualized using R (version 4.0.3). Comparisons between groups were made using the Chi‐square test for categorical variables and the Student's t‐test or Wilcoxon rank‐sum test for continuous variables. For the experimental data, GraphPad Prism 8 was used for statistical analysis and plotting. Values of *P* < 0.05 were considered statistically significant.

## Conflict of Interest

The authors declare no conflict of interest.

## Author Contributions

X.W., B.P., C.C., Y.Z., and J.M. contributed equally to this work. P.S., X.L., and W.C. performed conceptualization. X.W., B.P., C.C., Y.Z., J.M., Y.M., W.Z., H.Z., G.Z., S.L. and A.A. performed methodology. X.W., B.P., C.C., Y.Z., W.C., X. L, and P.S. performed investigation. X.W., B.P., C.C., and X.L. performed visualization. P.S. performed funding acquisition. W.C., X.L., and P.S. performed project administration. G.Z., S.L., W.C., X.L., and P.S. performed supervision. X.W, B.P., C.C., and X.L. wrote the original draft.

## Supporting information



Supporting Information

## Data Availability

The data that support the findings of this study are available from the corresponding author upon reasonable request.
