# Supplementary material for: CXCL16/CXCR6/TGF‐β Feedback Loop Between M‐MDSCs and Treg Inhibits Anti‐Bacterial Immunity During Biofilm Infection
Source: Adv Sci (Weinh). 2024 Dec 24;12(7):2409537. doi: 10.1002/advs.202409537 (PMC11831521; doi:10.1002/advs.202409537)

**Title**

CXCL16/CXCR6/TGF-β feedback loop between M-MDSCs and Treg inhibits anti-bacterial immunity during biofilm infection

**Supplementary Figure Legends**

**Fig S1. The single-cell landscape of PJI, AF and OA knee synovial tissues, related to Figure 1.**

a, The UMAP plot showed the projections of individual samples after correcting for batch effects by Harmony (PJI, n=6; AF, n=4; OA, n=3).

b, The bar chart showed the proportion of each cell type in different samples (PJI, n=6; AF, n=4; OA, n=3).

c, The bar chart showed the numbers of each cell type in PJI, AF and OA.

d, The bar chart showed the numbers of each cell type in different samples (PJI, n=6; AF, n=4; OA, n=3).

e, The heatmap showed top ten markers for each cluster.

f, The dot plot showed the expression level of biomarkers for major cell types.

**Fig S2. The biomarkers of M-MDSCs****, related to Figure 2.**

a, The bar chart showed the proportion of each myeloid subpopulation in PJI, AF and OA.

b, The dot plot showed the expression level of biomarkers for myeloid cell subpopulations.

c, The dot plot showed the expression level of biomarkers in M-MDSCs derived from monocytes.

d, The UMAP plot showed detailed annotation of the myeloid subpopulations merging with neutrophils after re-clustering.

e, The UMAP plot showed the expression level of representative biomarkers for neutrophils.

f, The dot plot showed the expression of immunosuppressive genes of M-MDSCs in PJI compared with AF and OA.

g, GSVA showed activation of immunosuppressive signaling pathways of M-MDSCs in PJI compared with AF and OA.

**Fig S3 The differentiation trajectory of synovial lymphocytes, related to Figure 3.**

a, The bar chart showed the proportion of each lymphocyte subpopulation in PJI, AF and OA.

b, The dot plot showed the expression level of biomarkers for lymphocyte subpopulation.

c, The UMAP plot showed pseudotime analysis of lymphocytes from synovial cells.

d, The UMAP plot showed the speculated pseudotime of the trajectory.

**Fig S4 The proportion of Treg in OA and RA of scRNA data from the database.**

a-d, The UMAP plot showed the biomarkers of T cells and Treg, and the distribution of Treg from RA.

e, The bar chart showed the proportion of Treg in RA.

f, The UMAP plot showed detailed annotation of the synovial cells from OA and RA.

g, The UMAP plot showed the expression level of biomarkers for major cell populations.

h, The bar chart showed the proportion of each cell type in different samples (OA, n=4; RA, n=20).

i, The bar chart showed the proportion of each cell type in OA and RA.

j-k, The UMAP plot showed the biomarkers and distribution of Treg from OA and RA.

l, The bar chart showed the proportion of Treg in different samples (OA, n=4; RA, n=20).

m, The bar chart showed the proportion of Treg in OA and RA.

n, The boxplot showed the compositional shifts comparing the proportion of Treg between OA and RA. Unpaired t test; ^ns^p < 0.05.

o, The UMAP plot showed detailed annotation of the synovial cells from OA (n=3).

p, The UMAP plot showed the expression level of biomarkers for major cell populations.

q-r, The UMAP plot showed the biomarker (CTLA4) of Treg.

s, Quantitative analysis of Treg. Unpaired t test; *p < 0.05, **p < 0.01.

**Fig S5 The fibroblast subpopulations exhibited the stratification of synovial cells.**

a, The UMAP plot showed detailed annotation of the fibroblast subpopulations after re-clustering. Four fibroblast clusters were visualized. Fibro Fibroblast.

b, The UMAP plot showed the expression level of biomarkers for fibroblast subpopulations.

c, The bar chart showed the proportion of each fibroblast subpopulation in PJI, AF and OA.

d, The UMAP plot showed pseudotime analysis of fibroblast from synovial cells.

e, The UMAP plot showed the speculated pseudotime of the trajectory.

f, The expression dynamics of selected marker genes (CXCL12, PRG4, TGFBI, THY1).

g, The dot plot showed the expression level of biomarkers for fibroblast subpopulations.

h, GSVA showed different enriched pathways of the four fibroblast subpopulations.

**Fig S6 The** **endothelial cell subpopulations exhibited the stratification of synovial cells.**

a, The UMAP plot showed detailed annotation of the endothelial cell subpopulations after re-clustering. Six endothelial cell clusters were visualized.

b, The UMAP plot showed the expression level of biomarkers for endothelial cell subpopulations.

c, The bar chart showed the proportion of each endothelial cell subpopulation in PJI, AF and OA.

d, The UMAP plot showed pseudotime analysis of endothelial cell from synovial cells.

e, The UMAP plot showed the speculated pseudotime of the trajectory.

f, The expression dynamics of selected marker genes (PGF, PXDN).

g, The dot plot showed the expression level of biomarkers for endothelial cell subpopulations.

h, GSVA showed different enriched pathways of the four endothelial cell subpopulations.

**Fig S7 The activation of CXCL signaling pathway, related to Figure 4.**

a-g, The circle plot showed the inferred intercellular communication network for all the ligand-receptor pairs of CXCL signaling pathway.

h, Violin plots showed the expression of CXCL16 among PJI, AF and OA in each myeloid subpopulation.

i, Violin plots showed the expression of CXCR6 among PJI, AF and OA in each lymphocyte subpopulation.

j, The expression of CXCL16 protein in two subtypes of MDSCs (M-MDSCs, n=5; PMN-MDSCs, n=5). Unpaired t test; ****p < 0.0001.

k, The expression of CXCL16 protein was determined following co-cultivation of M-MDSCs with *S. aureus* biofilm using ELISA (M-MDSCs, n=5; M-MDSCs+*S. aureus* biofilm, n=5). Unpaired t test; ****p < 0.0001.

**Fig S8 Constructing model of 3D-printing prosthesis PJI mice, related to Figure 6-7.**

a, The surgical field of NC and PJI mice models.

b, The roentgenograms were taken both at the knee extension and maximum flexio.

c, Representative H&E stained photomicrographs of knee joint sagittal sections of PJI mices at specified time points.

d-e, The process of constructing Treg-specific CXCR6 knockout mice.

f, The bar chart showed the proportion of each T cell subpopulation in PJI, AF and OA.

g, The dot plot showed the expression level of biomarkers for T cell subpopulations.

**Fig. S1**


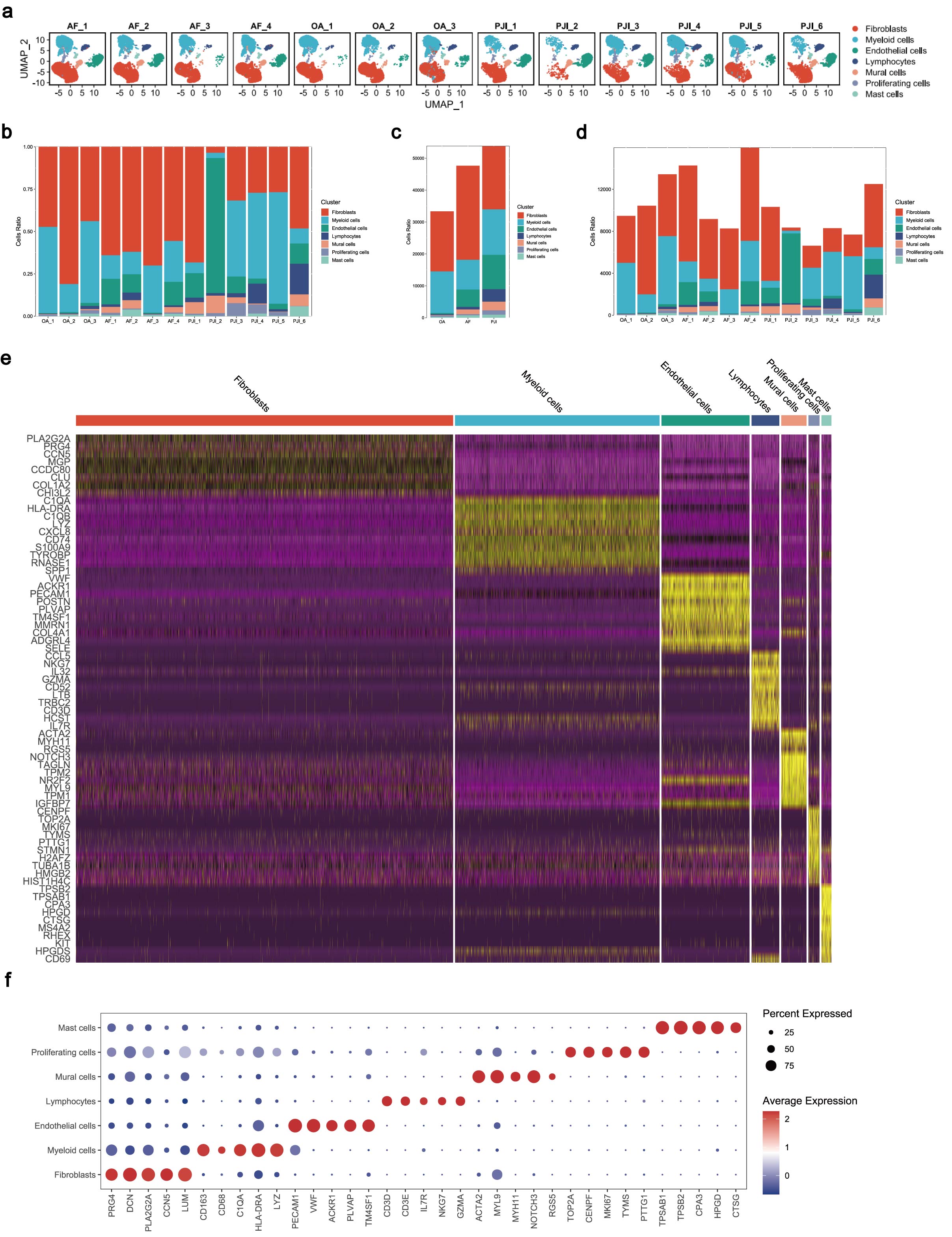


**Fig. S2**


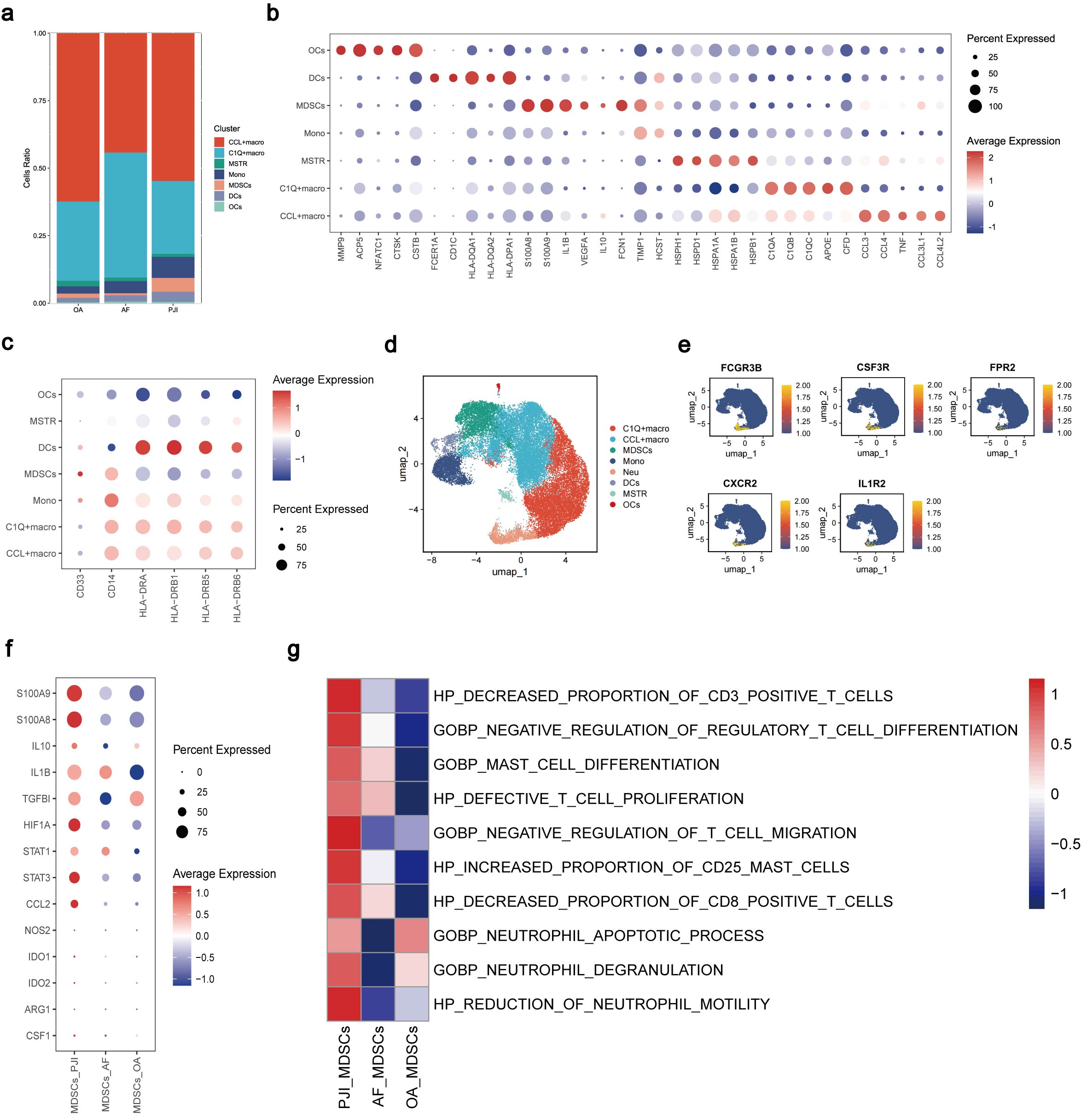


**Fig. S3**


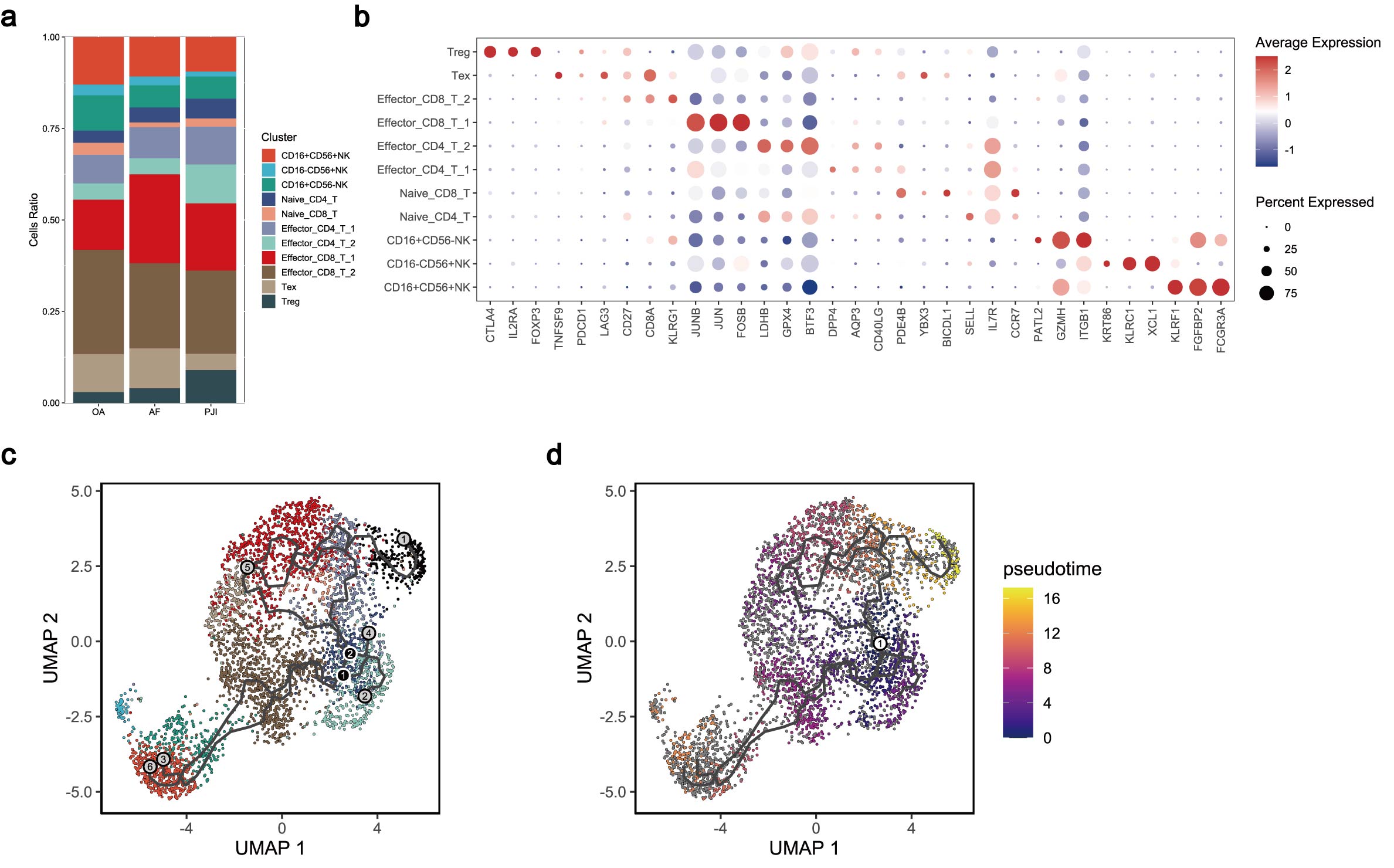


**Fig. S4**


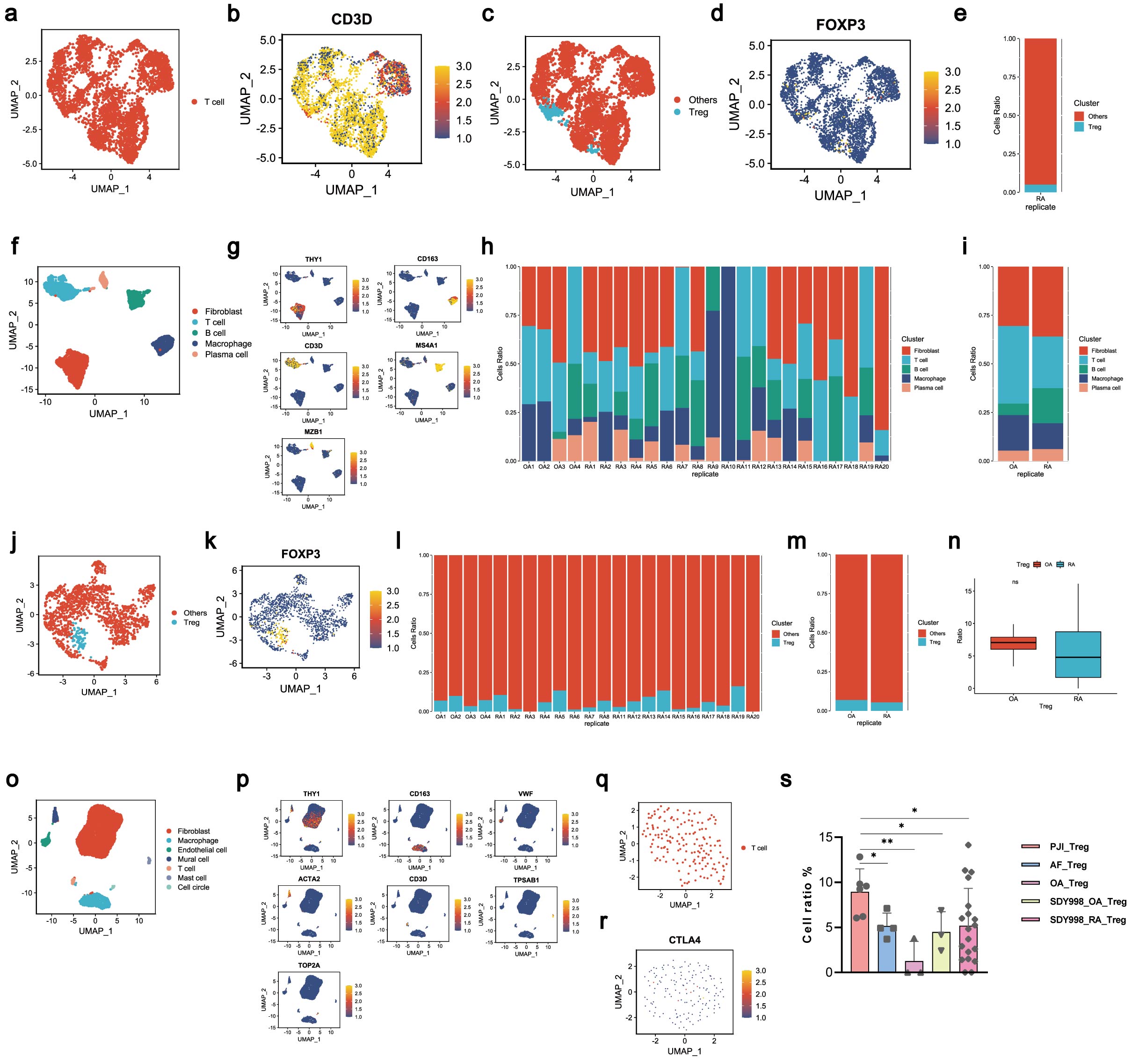


**Fig. S5**


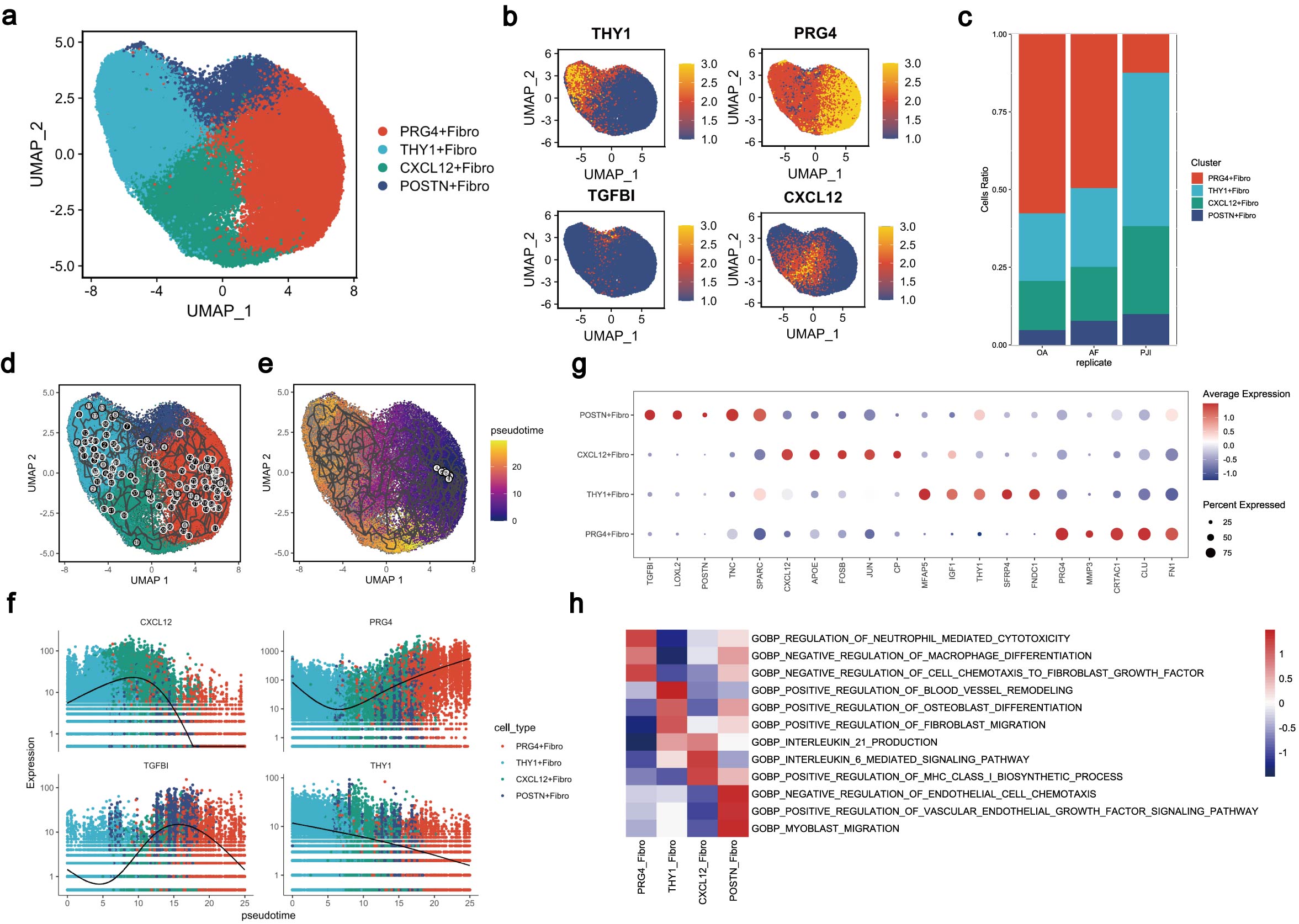


**Fig. S6**


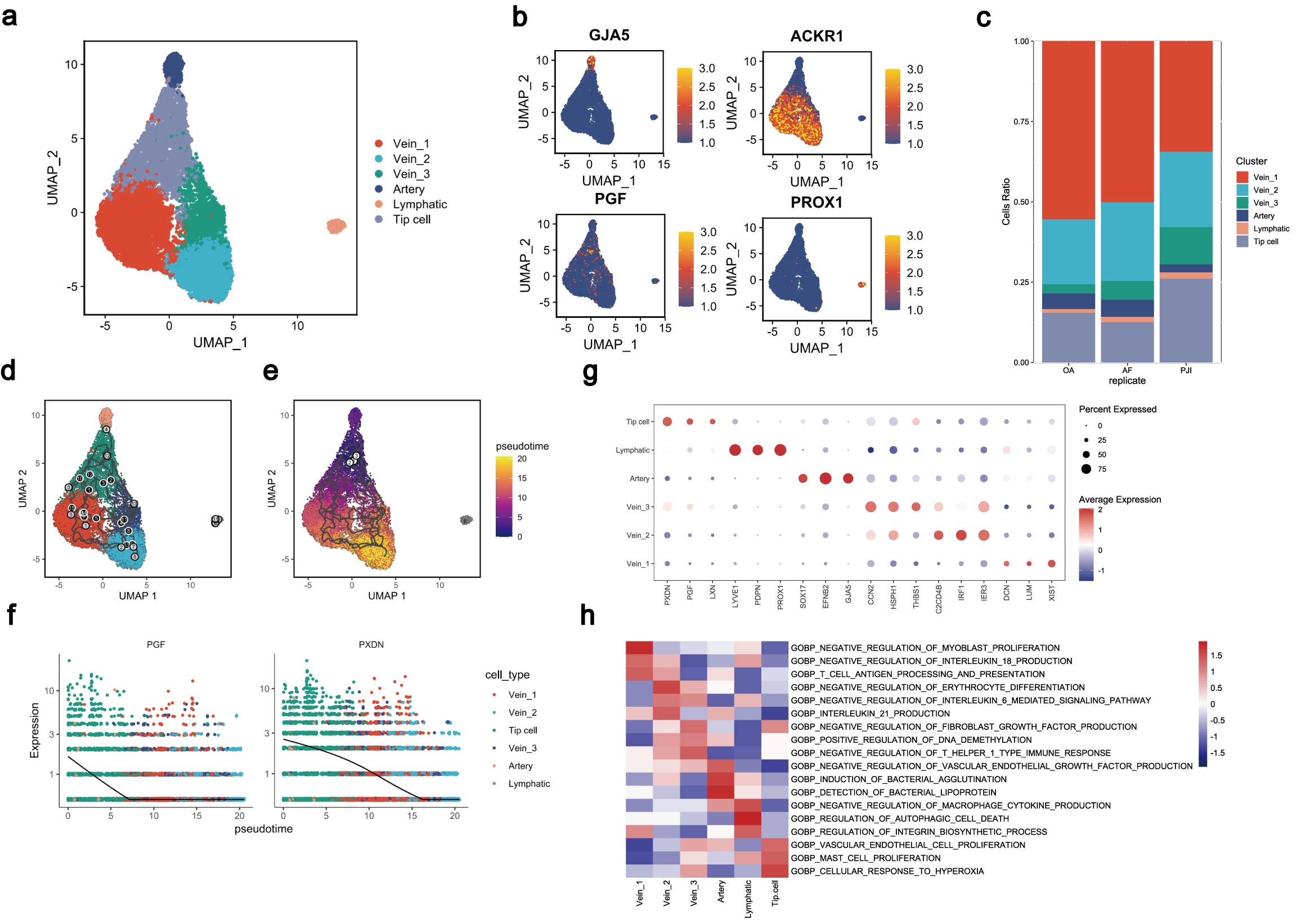


**Fig. S7**


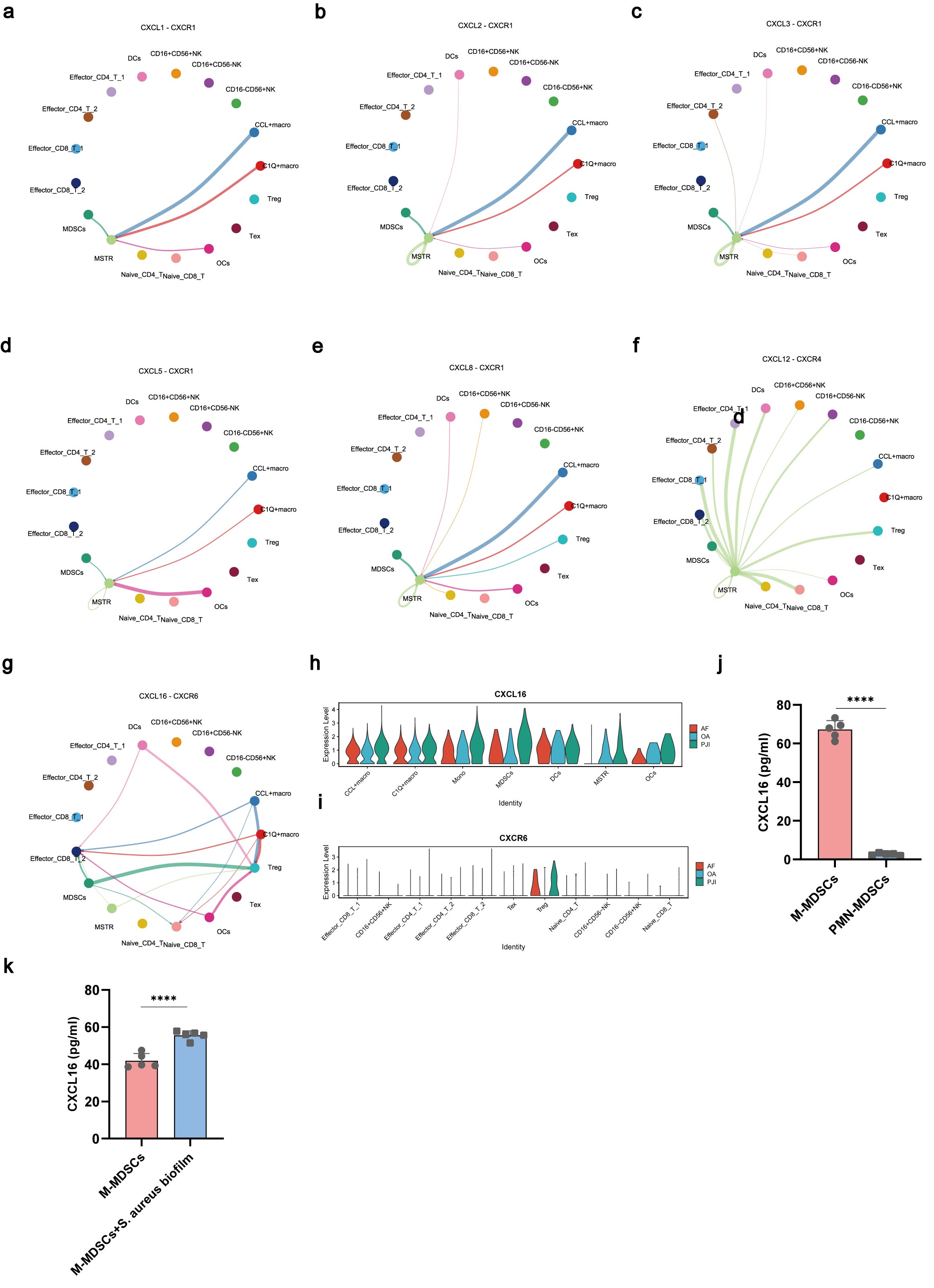


**Fig. S8**


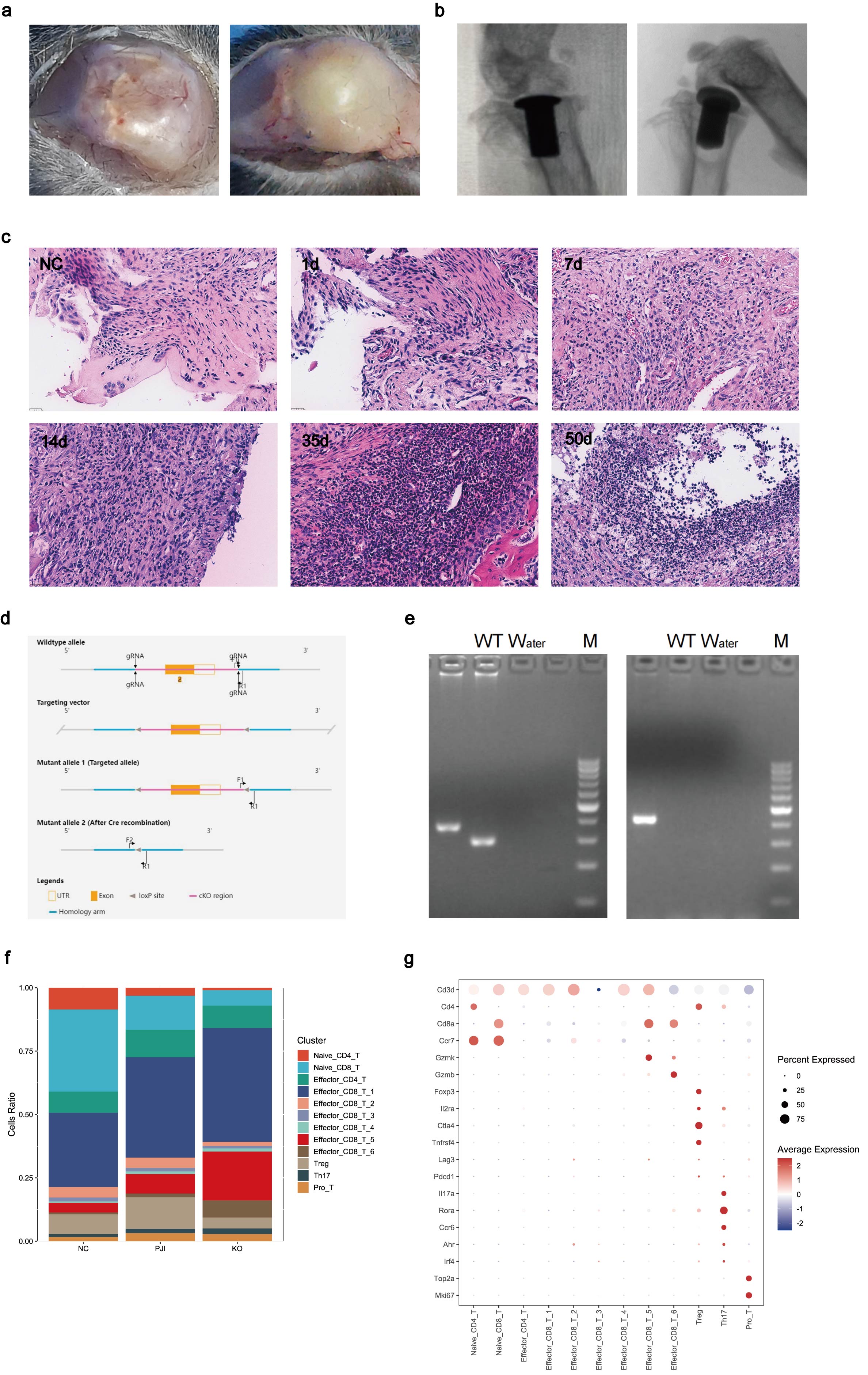

Supplement: Supplementary file 1 — Supporting Information [file ADVS-12-2409537-s001.docx]
